# DEAR1 Is a Dominant Regulator of Acinar Morphogenesis and an Independent Predictor of Local Recurrence-Free Survival in Early-Onset Breast Cancer

**DOI:** 10.1371/journal.pmed.1000068

**Published:** 2009-05-05

**Authors:** Steven T. Lott, Nanyue Chen, Dawn S. Chandler, Qifeng Yang, Luo Wang, Marivonne Rodriguez, Hongyan Xie, Seetharaman Balasenthil, Thomas A. Buchholz, Aysegul A. Sahin, Katrina Chaung, Baili Zhang, Shodimu-Emmanu Olufemi, Jinyun Chen, Henry Adams, Vimla Band, Adel K. El-Naggar, Marsha L. Frazier, Khandan Keyomarsi, Kelly K. Hunt, Subrata Sen, Bruce Haffty, Stephen M. Hewitt, Ralf Krahe, Ann McNeill Killary

**Affiliations:** 1 Department of Genetics, The University of Texas M. D. Anderson Cancer Center, Houston, Texas, United States of America; 2 Department of Radiation Oncology, University of Medicine & Dentistry of New Jersey–Robert Wood Johnson Medical School, New Brunswick, New Jersey, United States of America; 3 Department of Radiation Oncology, The University of Texas M. D. Anderson Cancer Center, Houston, Texas, United States of America; 4 Division of Pathology and Laboratory Medicine, The University of Texas M. D. Anderson Cancer Center, Houston, Texas, United States of America; 5 Department of Epidemiology, The University of Texas M.D. Anderson Cancer Center, Houston, Texas, United States of America; 6 Department of Genetics, Cell Biology and Anatomy, The University of Nebraska Medical Center, Eppley Cancer Center, Omaha, Nebraska, United States of America; 7 Department of Experimental Radiation Oncology, The University of Texas M. D. Anderson Cancer Center, Houston, Texas, United States of America; 8 Department of Surgical Oncology, The University of Texas M. D. Anderson Cancer Center, Houston, Texas, United States of America; 9 Tissue Array Research Program, Laboratory of Pathology, Center for Cancer Research, National Cancer Institute, National Institutes of Health, Bethesda, Maryland, United States of America; Univesity of Texas Southwestern Medical Center at Dallas, United States of America

## Abstract

**Background:**

Breast cancer in young women tends to have a natural history of aggressive disease for which rates of recurrence are higher than in breast cancers detected later in life. Little is known about the genetic pathways that underlie early-onset breast cancer. Here we report the discovery of *DEAR1* (*ductal epithelium–associated RING Chromosome 1*), a novel gene encoding a member of the TRIM (tripartite motif) subfamily of RING finger proteins, and provide evidence for its role as a dominant regulator of acinar morphogenesis in the mammary gland and as an independent predictor of local recurrence-free survival in early-onset breast cancer.

**Methods and Findings:**

Suppression subtractive hybridization identified *DEAR1* as a novel gene mapping to a region of high-frequency loss of heterozygosity (LOH) in a number of histologically diverse human cancers within Chromosome 1p35.1. In the breast epithelium, *DEAR1* expression is limited to the ductal and glandular epithelium and is down-regulated in transition to ductal carcinoma in situ (DCIS), an early histologic stage in breast tumorigenesis. *DEAR1* missense mutations and homozygous deletion (HD) were discovered in breast cancer cell lines and tumor samples. Introduction of the *DEAR1* wild type and not the missense mutant alleles to complement a mutation in a breast cancer cell line, derived from a 36-year-old female with invasive breast cancer, initiated acinar morphogenesis in three-dimensional (3D) basement membrane culture and restored tissue architecture reminiscent of normal acinar structures in the mammary gland in vivo. Stable knockdown of *DEAR1* in immortalized human mammary epithelial cells (HMECs) recapitulated the growth in 3D culture of breast cancer cell lines containing mutated *DEAR1*, in that sh*DEAR1* clones demonstrated disruption of tissue architecture, loss of apical basal polarity, diffuse apoptosis, and failure of lumen formation. Furthermore, immunohistochemical staining of a tissue microarray from a cohort of 123 young female breast cancer patients with a 20-year follow-up indicated that in early-onset breast cancer, *DEAR1* expression serves as an independent predictor of local recurrence-free survival and correlates significantly with strong family history of breast cancer and the triple-negative phenotype (ER^−^, PR^−^, HER-2^−^) of breast cancers with poor prognosis.

**Conclusions:**

Our data provide compelling evidence for the genetic alteration and loss of expression of *DEAR1* in breast cancer, for the functional role of *DEAR1* in the dominant regulation of acinar morphogenesis in 3D culture, and for the potential utility of an immunohistochemical assay for *DEAR1* expression as an independent prognostic marker for stratification of early-onset disease.

## Abstract 2

Ann Killary and colleagues describe a new gene that is genetically altered in breast tumors, and that may provide a new breast cancer prognostic marker.

## Editors' Summary

**Background:**

Each year, more than one million women discover that they have breast cancer. This type of cancer begins when cells in the breast that line the milk-producing glands or the tubes that take the milk to the nipples (glandular and ductal epithelial cells, respectively) acquire genetic changes that allow them to grow uncontrollably and to move around the body (metastasize). The uncontrolled division leads to the formation of a lump that can be detected by mammography (a breast X-ray) or by manual breast examination. Breast cancer is treated by surgical removal of the lump or, if the cancer has started to spread, by removal of the whole breast (mastectomy). Surgery is usually followed by radiotherapy or chemotherapy. These “adjuvant” therapies are designed to kill any remaining cancer cells but can make patients very ill. Generally speaking, the outlook for women with breast cancer is good. In the US, for example, nearly 90% of affected women are still alive five years after their diagnosis.

**Why Was This Study Done?:**

Although breast cancer is usually diagnosed in women in their 50s or 60s, some women develop breast cancer much earlier. In these women, the disease is often very aggressive. Compared to older women, young women with breast cancer have a lower overall survival rate and their cancer is more likely to recur locally or to metastasize. It would be useful to be able to recognize those younger women at the greatest risk of cancer recurrence so that they could be offered intensive surveillance and adjuvant therapy; those women at a lower risk could have gentler treatments. To achieve this type of “stratification,” the genetic changes that underlie breast cancer in young women need to be identified. In this study, the researchers discover a gene that is genetically altered (by mutations or deletion) in early-onset breast cancer and then investigate whether its expression can predict outcomes in women with this disease.

**What Did the Researchers Do and Find?:**

The researchers used “suppression subtractive hybridization” to identify a new gene in a region of human Chromosome 1 where loss of heterozygosity (LOH; a genetic alteration associated with cancer development) frequently occurs. They called the gene *DEAR1* (*ductal epithelium-associated RING Chromosome 1*) to indicate that it is expressed in ductal and glandular epithelial cells and encodes a “RING finger” protein (specifically, a subtype called a TRIM protein; RING finger proteins such as BRCA1 and BRCA2 have been implicated in early cancer development and in a large fraction of inherited breast cancers). DEAR1 expression was reduced or lost in several ductal carcinomas in situ (a local abnormality that can develop into breast cancer) and advanced breast cancers, the researchers report. Furthermore, many breast tumors carried *DEAR1* missense mutations (genetic changes that interfere with the normal function of the DEAR1 protein) or had lost both copies of *DEAR1* (the human genome contains two copies of most genes). To determine the function of DEAR1, the researchers replaced a normal copy of *DEAR1* into a breast cancer cell that had a mutation in *DEAR1*. They then examined the growth of these genetically manipulated cells in special three-dimensional cultures. The breast cancer cells without *DEAR1* grew rapidly without an organized structure while the breast cancer cells containing the introduced copy of *DEAR1* formed structures that resembled normal breast acini (sac-like structures that secrete milk). In normal human mammary epithelial cells, the researchers silenced *DEAR1* expression and also showed that without *DEAR1*, the normal mammary cells lost their ability to form proper acini. Finally, the researchers report that *DEAR1* expression (detected “immunohistochemically”) was frequently lost in women who had had early-onset breast cancer and that the loss of *DEAR1* expression correlated with reduced local recurrence-free survival, a strong family history of breast cancer and with a breast cancer subtype that has a poor outcome.

**What Do These Findings Mean?:**

These findings indicate that genetic alteration and loss of expression of *DEAR1* are common in breast cancer. Although laboratory experiments may not necessarily reflect what happens in people, the results from the three-dimensional culture of breast epithelial cells suggest that DEAR1 may regulate the normal acinar structure of the breast. Consequently, loss of *DEAR1* expression could be an early event in breast cancer development. Most importantly, the correlation between *DEAR1* expression and both local recurrence in early-onset breast cancer and a breast cancer subtype with a poor outcome suggests that it might be possible to use DEAR1 expression to identify women with early-onset breast cancer who have an increased risk of local recurrence so that they get the most appropriate treatment for their cancer.

**Additional Information:**

Please access these Web sites via the online version of this summary at http://dx.doi.org/10.1371/journal.pmed.1000068.

- This study is further discussed in a *PLoS Medicine* Perspective by Senthil Muthuswamy
- The US National Cancer Institute provides detailed information for patients and health professionals on all aspects of breast cancer, including information on genetic alterations in breast cancer (in English and Spanish)
- The MedlinePlus Encyclopedia provides information for patients about breast cancer; MedlinePlus also provides links to many other breast cancer resources (in English and Spanish)
- The UK charities Cancerbackup (now merged with MacMillan Cancer Support) and Cancer Research UK also provide detailed information about breast cancer

## Introduction

Breast cancer is the most common cause of cancer-related death in women with an early onset of the disease (≤45 years of age) [1]. Although breast cancer occurs less frequently in young women than in older women, it is often associated with a poorer prognosis. Compared with older women, young women with breast cancer have decreased overall survival and disease-free survival rates, and a higher percentage of tumors with pathologic features reflective of aggressive disease [2]–[6]. In early onset breast cancers without nodal involvement, approximately one-fourth will recur up to 12 years postsurgery [6]. In addition, younger age is recognized as a risk factor for local–regional recurrence and for distant metastases after either breast conservation treatment or mastectomy [5],[6]. Biomarkers are urgently needed to identify young women who have an increased risk of breast cancer recurrence and would therefore benefit from heightened surveillance and adjuvant therapy. However, in order to stratify early-onset cancers, the genetic mechanisms that underlie breast cancer in young women must first be elucidated.

The initiation and progression of breast cancer is thought to involve not only a disruption of cellular pathways that underlie proliferation, differentiation, and death, but also perturbation of extracellular signaling pathways that influence differentiation and tissue architecture. The architecture of the human mammary gland is an elaborate branched ductal lobular network terminating in individual acinar units composed of an inner layer of polarized, luminal epithelial cells surrounding a hollow lumen and an outer layer of myoepithelial cells separated from the stroma by an intact basement membrane [7]. Concomitant with initiation of tumorigenesis, the mammary gland loses tissue polarity and increases cellular proliferation [8]. Cell growth, differentiation, and death in the mammary epithelium are therefore in an intricate balance, the regulation of which is, at least in part, governed by microenvironmental signals from the extracellular matrix (ECM) [9].

Experimental modeling of the ECM using 3D basement membrane culture recapitulates the architecture of mammary ductal epithelium in vitro [9]–[11]. Human mammary epithelial cells (HMECs) as well as the immortalized mammary epithelial cell line MCF10A form polarized, growth-arrested acini in 3D culture [11],[12]. In sharp contrast, breast tumor cell lines propagated in 3D culture form nonpolarized clusters of cells without acinar formation and with limited differentiation [9]. Utilization of the 3D culture system has elucidated the importance of ECM signaling in the control of differentiation and in the initiation and progression of breast tumorigenesis [9]–[17]. Manipulation of the extracellular milieu by activation of key ECM signaling pathways results in the loss of differentiation associated with malignant progression [11]–[13]. Likewise, partial or complete restoration of acinar formation in breast cancer cell lines grown in 3D culture has also been documented [14]–[16]. Phenotypic restoration of acinar morphogenesis in 3D culture was observed irrespective of the accumulation of genetic alterations in the tumor cells, suggesting that the differentiation state in the breast epithelium is in a dynamic state, amenable to therapeutic intervention in the case of breast cancer, and that the regulation imposed by the ECM is dominant to tumor-specific mutational events in the control of breast cancer progression.

However, RNAi-mediated knockdown of BRCA1 in MCF10A cells results in a failure of acinus formation and increase in proliferation in 3D culture, suggesting that critical genes that are mutated in human breast cancer could function in the dominant regulation of acinar morphogenesis and differentiation in the mammary epithelium [17]. Here we report the discovery of *DEAR1*, a novel gene that undergoes genetic alteration in breast cancer, and the investigation of *DEAR1*'s role in regulation of acinar morphogenesis and its potential to aid in the clinical management of early-onset disease.

## Materials and Methods

### Cell Lines and Tumor Samples

The 21NT, 21PT, and 21MT lines were propagated in Dulbecco's modified Eagle medium/F12 (DMEM/F12) supplemented with 10% fetal bovine serum, 1 µg/ml insulin, 12.5 µg/ml epidermal growth factor, and 1 µg/ml hydrocortisone. HMECs (ATCC, Manassas, Virginia, United States) and the immortalized breast epithelial line MCF10A were propagated in MEGM medium (Clontech Laboratories, Mountain View, California, United States) according to ATCC protocols. The remainder of the breast carcinoma cell lines (T47D, BT474, MCF-7, H38, Zr75T, MDA-MB-157, HBL100, HS578T, BT20T, MDA-MB-231, and MDA-MB-436) used for mutation screens and expression studies were grown in DMEM/F12 supplemented with 10% fetal bovine serum.

### PCR Select Subtractive Hybridization

Total RNA was isolated with TRIzol (Invitrogen, Carlsbad, California, United States) with subsequent isolation of the poly-A^+^ population using oligo dT cellulose. The PCR-Select suppression subtractive hybridization assay (Clontech Laboratories, Palo Alto, California, United States) was used to identify cDNAs differentially expressed between the microcell hybrid lines SN19(3)EEE [driver] and SN19(3i)YY [tester] [18]–[21] (as described in Figure S1 and Text S1). PCR products from the secondary PCR reactions were cloned into the pCRII vector (Invitrogen).

### Library Screening

To identify a full-length cDNA clone of *DEAR1*, a human retinoic acid induced neuroepithelial cell library cloned into the ZAP Express XR vector (Stratagene, La Jolla, California, United States) was screened with a ^32^P end-labeled oligonucleotide corresponding to the 5′ end of the partial cDNA (*DEAR1*-FOR, 5′-TTGATCCAAGGATGTGACATG-3′). Positive plaques were excised and confirmed by PCR using *DEAR1*-FOR with *DEAR1*-REV (5′-GTGACCACTGTGGACTGGG-3′). The ExAssist helper phage was used to excise the pBK-CMV expression vector–positive ZAP Express clones according to the manufacturer's protocol. Sequencing of this phagemid identified an alternative splice form of *DEAR1* (exons 1–3 and 5). Screening of the RPCI 4 PAC library (Children's Hospital Oakland Research Institute, http://bacpac.chori.org/) using the phagemid insert end-labeled with ^32^P was performed to identify a genomic clone of *DEAR1*.

### Generation of a Full-Length Expression Construct

To obtain a cDNA with all exons, one through five, the IMAGE clone 3355572 was obtained from ATCC. Using this clone as a template, the open reading frame was amplified by PCR (forward primer, M13, 5′-GTAAAACGACGGCCAGT-3′; reverse primer, 7b5-2032-AS, 5′-GTCTTAGGCCATGGGACATAAGAG-3′). This amplification yielded a 1,972 bp product that was subsequently ligated into pBK-CMV digested with EcoRI/XhoI.

### FISH Mapping

FISH mapping of PAC clones was performed according to previously published protocols [18].

### Promoter Methylation and Deletion Analysis

Pyrosequencing-based methylation analysis (PMA) was performed according to the method of Colella et al. [22]. Primers for deletion studies and promoter analysis by PMA are available upon request.

### Mutation Screening

For exons two through five, 100 ng of genomic DNA was amplified with AmpliTaq Gold Taq polymerase (Applied Biosystems, Foster City, California, USA). Since the amplification of exon 1 proved difficult and inconsistent under standard conditions (due in part to its G-C content), alternative conditions were used [23]. The intronic primer sequences are as follows: exon 1 forward, 5′-GCTCCTACCCCTGCCTGT-3′ and exon 1 reverse, 5′-CCCCACCTCCAGCCC-3′; exon 2 forward, 5′-GCAGTGGTCAGGGCTGAATG-3′ and exon 2 reverse, 5′-CCTTCTTCCCCAGCTGGC-3′; exon 3 forward, 5′-CTGTGGTGTCAAGGCTCTCGA-3′ and exon 3 reverse, 5′-CTCTGCTAAGGATCCCATCTG-3′; exon 4 forward 5′-CACATCCTATGCCAGCTGC-3′ and exon 4 reverse, 5′-CAAGGCACTCAGCACATTC-3′; exon 5 forward, 5′-CTGGAAGGACCTTAACCACCA-3′, and exon 5 reverse 5′-CTATCTTCCGGGCAGGGCTC-3′. The expected product sizes are: 585 bp for exon 1, 250 bp for exon 2, 420 bp for exon 3, 329 bp for exon 4 and 800 bp for exon 5. PCR products were treated with ExoSAP (USB, Cleveland, Ohio, United States) and submitted to the M. D. Anderson DNA core facility for sequencing or denaturing high-performance liquid chromatography (DHPLC). Electropherograms were analyzed using either Sequencher or Lasergene software packages.

### Antibody Production

Lasergene sequence analysis software was utilized to identify nonconserved regions of *DEAR1* that also scored highly for antigenicity. Peptide synthesis and polyclonal antibody production was performed by Bethyl Laboratories (Montgomery, Texas, United States). Rabbits were immunized with the *DEAR1* peptide conjugated to keyhole limpet hemocyanin. *DEAR1* antibodies were affinity-purified.

### Transient Transfection, Whole Cell Extracts, and Western Blotting

For detection of exogenous HA-DEAR1, 293T cells were seeded in a 24-well plate at 4×10^4^ cells/well overnight before transfection. To each well, 0.2 µg of pCMV-HA/*DEAR1* plasmid and FuGene6 transfection reagent (1 µg∶3 µl) (Roche Applied Science, Indianapolis, Indiana, United States) were added. After 24 h of culture, the cells were scraped into 60 µl of 1× SDS sample buffer. For whole cell lysates, cell lines were grown exponentially, harvested, and lysed in 1× SDS sample buffer. Equal amounts of protein per lane were loaded on 4%–20% SDS–PAGE gradient gels (Pierce, Rockford, Illinois, United States), transferred to membranes, and analyzed using antibodies against DEAR1 and β-actin (Sigma, Saint Louis, Missouri, United States). For peptide-blocking experiment, DEAR1 antibody was mixed with 5× peptide of DEAR1 (v/v) for 2 h at room temperature, prior to incubation with membrane.

### Stable Transfection

Transfection of the pBK-CMV/Δ187*DEAR1* and the pBK-CMV/*DEAR1* constructs into 21MT cells was performed using Lipofectamine 2000 (Invitrogen). Stable transfectants were isolated as single colonies following selection in G418 (500 µg/ml).

### DEAR1 Stable Knockdown

MISSION shRNA lentiviral vectors expressing nontarget control shRNA or *DEAR1* shRNAs and packaging vectors were purchased from Sigma (NM_018207). Cotransfection of retroviral and packaging vectors into HEK293T packaging cells for production and packaging of retroviruses was performed according to the manufacturer's recommendations. The supernatant containing virus was harvested and filtered 48–72 h after transfection. For infection, viral supernatant was added to 76N-E6 cells in the presence of 8 µg/ml hexadimethrine bromide. Stable clones were selected using puromycin (2 µg/ml).

### Three-Dimensional Culture

Three-dimensional culture assays for acinus formation were performed as described by Debnath et al. [24].

### Immunohistochemistry

Immunohistochemistry was performed on 5 µm sections cut from formalin-fixed, paraffin-embedded tissue. Following deparaffinization and rehydration, sections were subjected to antigen retrieval in either 10 mM sodium citrate, pH 6.0, for 15 min in a microwave pressure cooker for the *DEAR1* antibody, or incubation with protease XXIV (BioGenex, San Ramon, California, United States) for 10 min at room temperature for the α-laminin-5 antibody (Millipore, Billerica, Massachusetts, United States). Subsequent staining procedures were performed according to the Super Sensitive Non-Biotin HRP/DAB Detection System (BioGenex) with the primary antibodies diluted 1∶200 in Common Antibody Diluent (BioGenex). Sections were counterstained with Mayer's hematoxylin and mounted with Permount (Fisher Scientific, Pittsburg, Pennsylvania, United States). Human tissue was obtained with appropriate institutional review board approval. *DEAR1* expression was scored as negative expression when there was no detectable staining and positive expression when the staining was diffuse positive, focal positive, or strong positive.

### Statistical Analysis

A database containing *DEAR1* status and relevant covariables was assembled and analyzed using SAS Version 9.1 (SAS Institute, Cary, North Carolina, United States). All tests of statistical significance were two-sided. *p*-Values less than 0.05 were considered statistically significant. Bivariate analyses for the association between covariables and DEAR1 status included the Chi-square and Fisher's exact tests. Bivariate analyses for the associations between predictor variables and local and distant recurrence, and overall survival were conducted using the Kaplan Meier log-rank test and the Chi-square test for linear trend. In the multivariate analysis, DEAR1 proportional hazards regression determined significant predictors of disease-free survival and overall survival at a *p* = 0.05 level in the final model.

## Results

### *DEAR1* Is a Novel RBCC/TRIM Family Member Mapping to a Region of LOH in Breast Cancer within Chromosome 1p35.1

**Figure 1:**
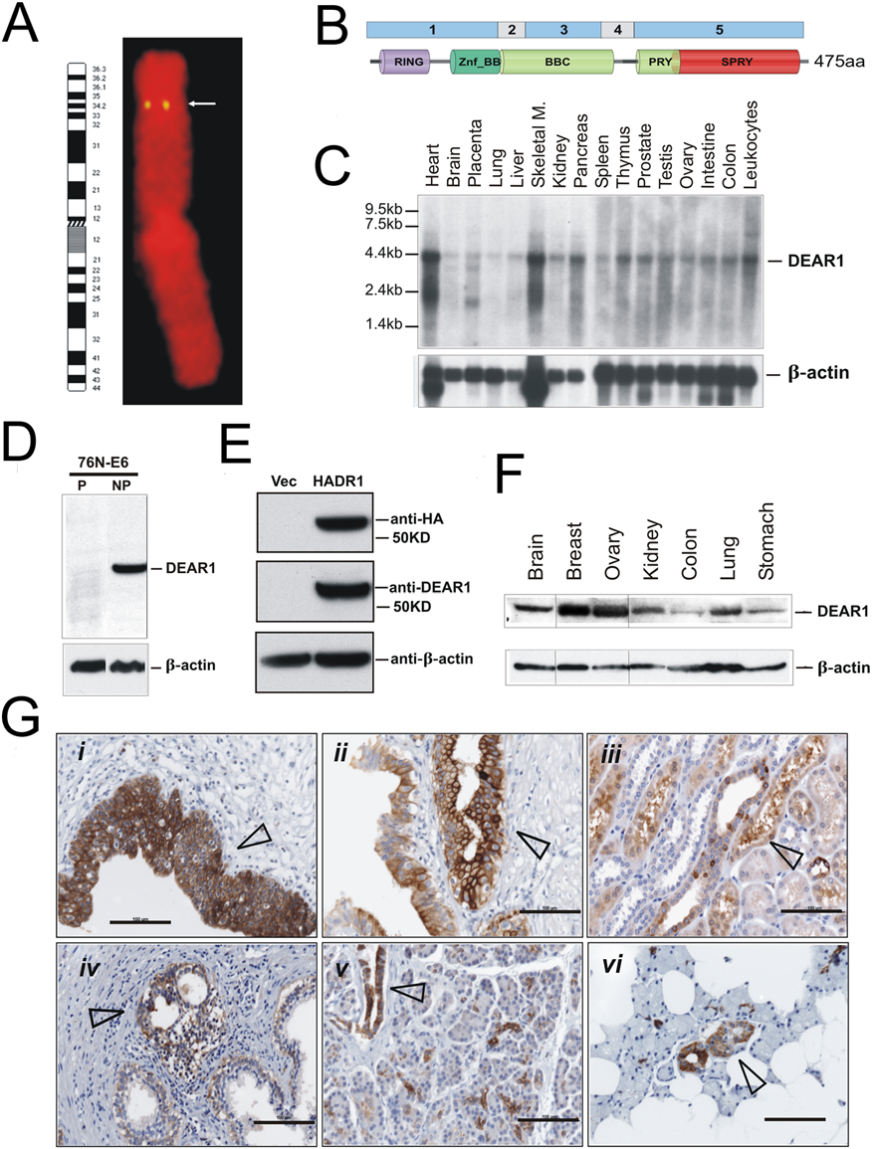
DEAR1 structure, mapping, and expression in normal tissues. (A) Chromosomal localization of *DEAR1* as determined by FISH analysis using the *DEAR1* P1-derived artificial chromosome (PAC) clone. (B) Graphical representation of DEAR1 exonic and protein structure. (C) DEAR1 multiple tissue Northern analysis detects a predominant 4.4 kb band in all tissues examined. Additional, lower molecular weight bands were observed in a number of tissues, including heart, placenta, skeletal muscle and brain. (D) DEAR1 peptide competition with 5× peptide specifically detects the predicted 54 kDa full-length protein in the immortalized HMEC line 76N-E6. (E) Transient transfection of HA-tagged DEAR1 into 293T cells (which do not express endogenous *DEAR1*) detects the appropriate sized protein. (F) Western blot analysis of normal tissue protein lysates using the α-N DEAR1 antibody identifies a strong band of approximately 54 kDa corresponding to the predicted full-length DEAR1 protein molecular weight. (G) Localization of DEAR1 protein in normal tissue assessed by immunohistochemistry using the α-N DEAR1 antibody on a multiple tissue microarray. Staining (dark brown, identified by arrow) is plainly visible in epithelial cells found in a wide range of tissues, including (*i*) bladder, (*ii*) gall bladder, (*iii*) kidney, (*iv*) prostate, (*v*) pancreas, and (*vi*) salivary gland.

One of the most studied genomic intervals in human cancer lies within the short arm of human Chromosome 1 in which LOH, within three separate intervals, occurs at high frequency in a variety of epithelial cancers, including both sporadic breast cancers and breast cancers with inherited predisposition [25]–[28]. LOH within Chromosome 1p has been shown to predict poor prognosis in node-negative breast cancers, and allelic deletions in the 1p36 and 1p32 region have been found to correlate with poor survival [28]. In screening cDNAs obtained from a suppression subtractive hybridization library (Figure S1 and Text S1), we identified a 700 bp partial cDNA with significant sequence similarity to a family of RING finger proteins (3.9×10^−18^) and that mapped by fluorescence in situ hybridization (FISH) to one of the Chromosome 1p genomic intervals with LOH in breast cancer within Chromosome 1p34-35. Subsequent screening of the human reference sequence (UCSC version hg18, based on NCBI Build 36 using the BLAT tool on the UCSC Genome Bioinformatics Web site, http://genome.ucsc.edu) unambiguously mapped the novel gene, *DEAR1* (*ductal epithelium–associated RING Chromosome 1*), to human Chromosome 1p35.1 (Figures 1A and S2).

The complete *DEAR1* open reading frame was identified by sequencing of additional cDNAs obtained from an NT2 neuroepithelial cDNA library screen and reverse transcription PCR of placental RNA. *DEAR1* is composed of five exons encoding a 475 amino acid protein with a predicted tripartite sequence motif associated with the RBCC (RING-B-box-Coiled-Coil)/TRIM (tripartite motif) subfamily of RING finger proteins with PRY and SPRY domains present within the carboxyl terminus (Figure 1B) [29]–[35]. The RBCC/TRIM family of RING finger proteins has been shown to play critical roles in the formation and architecture of multiprotein complexes within both the cytoplasm and nucleus, and has been implicated in the regulation of differentiation, development, and oncogenesis in multiple cell types and species [29]–[31]. Human family members have been associated with initiating events in oncogenesis, due to either loss of growth/tumor suppressor functions (*PML*) or gain of oncogenic functions (*RFP, Efp*) in addition to participating as oncogenic fusion partners in specific chromosomal translocation events, such as *PML, TIF1,* and *RFP* [33]–[36]. BLAST comparisons to the nonredundant peptide sequence databases indicated that *DEAR1* was a unique member of the RBCC/TRIM gene family (hypothetical protein FLJ10759, later annotated as TRIM 62) with the closest similarity to other human RBCC/TRIM family members being a 29% identity with the human Ret finger protein RFP, originally identified as a fusion partner with the RET tyrosine kinase proto-oncogene [33]. *DEAR1* is essentially identical (98%) to mouse and rat sequences (NP_835211 [*Mus musculus*] and XP_232757 [*Rattus norvegicus*]) (Figure S3) as well as RBCC/TRIM proteins from diverse species, including *Xenopus laevis* XNF7 (33% identity) and TRIM39 (32% identity in mouse, rat, and human) [32],[33].

### *DEAR1* Expression Is Limited to the Ductal and Glandular Epithelium in Normal Tissues

*DEAR1* is detected as a 4.4 kb primary transcript in multiple tissues on Northern analysis, with other smaller transcripts expressed in either a developmental or tissue-specific pattern in skeletal muscle, placenta, brain, and heart (Figure 1C). Affinity purified anti-peptide antibodies were generated to the amino terminus of the DEAR1 protein. Peptide blocking experiments, performed in HMECs to confirm the specificity of the novel antibody, indicated that the amino-terminal DEAR1 antibody detects the predicted 54 kDa full-length protein and that binding is specifically competed away in the presence of excess DEAR1 peptide (Figure 1D). In addition, transient transfection assays using HA-tagged *DEAR1* constructs introduced into 293T cells specifically detected the appropriate sized transcript (Figure 1E). Western analysis confirmed that *DEAR1* is expressed in all normal tissues analyzed (Figure 1F). However, *DEAR1* expression is localized to the ductal and glandular epithelium. Immunohistochemical analysis of a normal-tissue microarray (Biogenex) detected *DEAR1* expression limited to the epithelial lining of the ducts and glands in the majority of normal tissues examined, including bladder, gall bladder, kidney, prostate, pancreas, and salivary gland (Figure 1G [*i–vi*, respectively]).

### DEAR1 Expression Is Down-regulated in Breast Carcinoma Cell Lines and in Transition to Ductal Carcinoma In Situ

**Figure 2:**
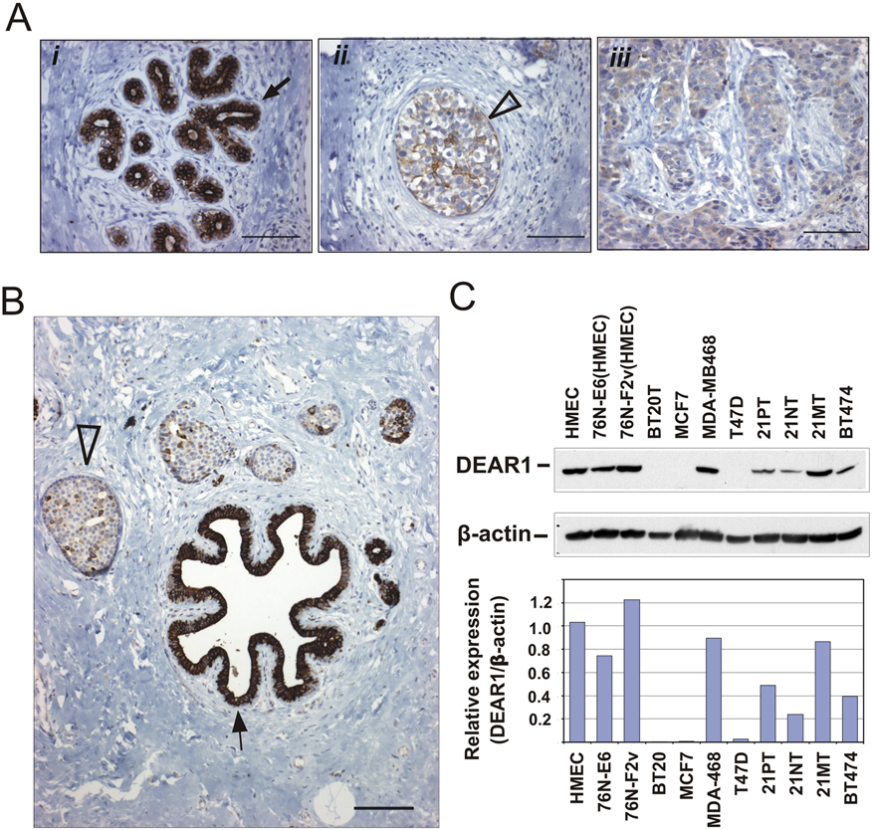
Down-regulation of DEAR1 in breast cancer cell lines and in transition to DCIS in the breast epithelium. (A) and (B) show immunohistochemical staining of two examples from 14 cases for which normal ductal structures, DCIS, and invasive carcinoma from the same individual are located within the same histologic section. Normal ducts are indicated by solid arrows, and representative foci of DCIS are indicated by an open arrowhead. Immunohistochemical staining using the α-N DEAR1 antibody appears as a dark brown precipitate. Panel (A) indicates (*i*) intense staining of DEAR1 in normal mammary ducts; (*ii*) diffuse, low level staining of DEAR1 observed in this single focus of DCIS. Note the slight increase in DEAR1 staining toward the center of the focus; (*iii*) diffuse, low level staining of DEAR1 is observed throughout much of this region composed of invasive carcinoma. Panel (B) shows intense staining of DEAR1 noted in the normal duct, with a dramatic decrease in expression in adjacent foci of DCIS. (C) DEAR1 expression on Western blot analysis of HMEC cultures (normal HMECs and immortalized HMECs 76N-E6 and 76N-F2v) and breast carcinoma cell lines.

*DEAR1* expression was examined by immunohistochemistry on a series of 14 DCIS samples with associated adjacent normal epithelium and the corresponding invasive cancer from the same individual. High levels of staining were observed in normal mammary ductal structures consistent with normal tissue microarray data (Figure 2A [*i*] and 2B). However, 10/14 (71%) specimens showed loss or down-regulation of *DEAR1* expression in the transition from normal epithelium to DCIS (Figure 2A and 2B). In high-grade DCIS, *DEAR1* expression was diminished at the basement membrane, with focal positivity in the center of the DCIS lesions (Figure 2A [*ii*]). In specimens demonstrating down-regulation of *DEAR1* in DCIS transition, 5/10 specimens (50%, for which invasive carcinoma was available for analysis) showed loss or down-regulation in the adjacent invasive carcinoma (Figure 2A [*iii*]) with the remaining five of ten invasive lesions positive for DEAR1 staining (unpublished data). *DEAR1* expression was also examined in normal HMECs, immortal HMEC variants, and breast carcinoma cell lines by Western blot analysis. Results indicated that DEAR1 expression was absent or down-regulated in six of eight (75%) breast carcinoma cell lines including two of three 21T series cell lines derived from a 36-year-old female with infiltrating ductal adenocarcinoma as compared with normal or immortalized HMECs (Figure 2C) [37],[38].

### *DEAR1* Is Mutated and Deleted in Breast Cancer

**Figure 3:**
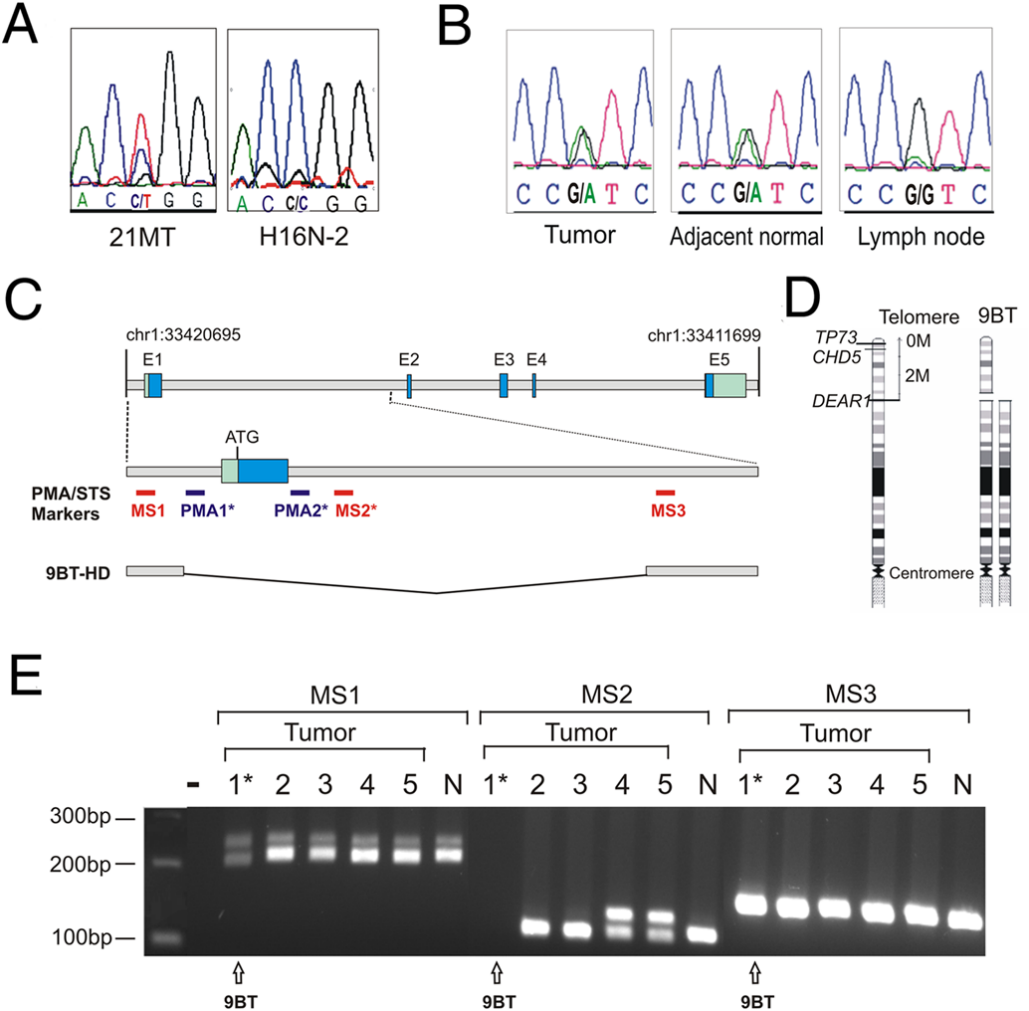
Mutation and microdeletion analysis of *DEAR1*. (A) Direct genomic sequencing identified a codon 187 missense mutation (C→T) in exon 3 in the 21MT cell line but not in the cell line H16N-2, derived from the normal mammary epithelium from the same patient. (B) A missense mutation in codon 473 of exon 5 (GTC→ATC, V473I) detected in a breast tumor sample as well as adjacent normal tissue, but not in the normal lymph node from this individual, indicating that the sequence alteration in the tumor was a somatic mutation of the *DEAR1* sequence. (C) Diagram of genomic structure and core promoter and exon 1 of *DEAR1* indicating the location of assays and primers by which HD in tumor 9BT was identified (indicated by *) as well as those used for deletion mapping in *DEAR1* and flanking genes. (D) Schematic of homozygous deletion in 9BT. (E) STS mapping analysis indicates retention of MS1, deletion of MS2, and retention of MS3 in primary tumor sample (9BT).

Mutational analysis was conducted on 12 breast cancer cell lines (itemized in Materials and Methods) and three cell lines of the 21T series (21NT, 21PT, and 21MT) by DHPLC and direct sequencing. All of the cell lines in the 21T series contained identical nonconservative missense mutations in exon 3 within codon 187 (CGG→TGG, R187W) in the coiled-coil domain not observed in 136 normal alleles or the SNP database (Figure 3A, Table S1). The mammary epithelial cell strain (H16N-2) derived from normal breast epithelium of the same patient as the 21T series lines, did not contain the codon 187 mutation, indicating that the genetic alteration in the 21T series is not a rare polymorphism, but rather a tumor-derived mutational event (Figure 3A). The R187W mutation falls between the two coils of the coiled-coil domain based on Parcoil (http://paircoil.lcs.mit.edu/cgi-bin/paircoil) and therefore might be predicted to affect protein binding to DEAR1. The mutation, however, does not affect protein stability following cycloheximide treatment (Figure S4). In addition to the 21T series mutations, breast cancer cell line MDA-MB-468 contained an intronic alteration not observed in the SNP database or in control lymphocytes (Table S1).

Sequence analysis of 55 primary breast tumors obtained from The University of Texas M. D. Anderson Cancer tumor bank revealed that 13% contained genetic alterations in *DEAR1,* including three missense mutations, three intronic alterations, and a silent mutation not observed in screening controls or the SNP database (Table S2). One missense mutation was observed in a breast tumor derived from a 36-year-old female, occurring one nucleotide downstream of the 21MT mutation and thereby altering the same codon 187 (CGG→CAG, R187Q) as the 21MT cell line mutation (Table S2). This mutation was observed in adjacent tissue but not in 136 normal alleles or the SNP database. Two missense mutations were identified in later-onset breast tumor samples, both affecting exon 5 (GTC→ATC, V473I and GTC→ATC, V350I) (Table S2) and present in both tumor and adjacent normal samples but not in controls or the SNP database. In addition, the exon 5 mutation was not observed in normal lymph node from the same individual whose tumor contained the codon 473 mutation, indicating that the sequence alteration in the tumor was a somatic mutation of the *DEAR1* sequence (Figure 3B). We also identified a HD in a primary tumor (9BT) obtained from a 39-year-old with triple-negative breast cancer. The deletion maps within the core promoter region of *DEAR1* using PMA (assays 1 and 2, Table S3) on bisulfite-treated DNA (Figure 3C and 3D). Genomic PCR confirmed the HD using STS markers that spanned microsatellite sequences MS1 and MS2, located upstream of the *DEAR1* core promoter and in the first intron, respectively (Figure 3C and 3D; Table S3). Results indicated that the MS1 region upstream of the 5′ UTR was retained in the 9BT sample (Figure 3D), thus mapping the breakpoint distal to MS1 and spanning the region identified by PMA. The distal boundary of the deletion was identified using primers that detected microsatellite sequence MS3 downstream of MS2 in intron 1, indicating that the HD encompassed the promoter and exon 1 with retention of exon 2 (MS3). Subsequent PMA detected a deletion of both the *CHD5* and *p73* genes, which lie distal to *DEAR1* in Chromosome 1p, suggestive of a terminal deletion of one allele with a breakpoint within the *DEAR1* promoter, which then resulted in LOH encompassing two distal candidate tumor suppressors on Chromosome 1p (Figure 3E). Importantly, the HD was detected by two separate methodologies, indicating a breakpoint in both alleles within the *DEAR1* promoter region. Thus, within a region of LOH for breast cancer and multiple epithelial tumors, we have identified a HD in an early-onset breast tumor. Additionally, because PMA detected heterozygous deletion of distal genes to *DEAR1*, and genomic PCR detected the HD limited to the *DEAR1* promoter and exon 1, our results are consistent with a microdeletion in one allele and a terminal deletion with a breakpoint in the promoter of *DEAR1* in the second allele, thereby deleting the entire *DEAR1* coding region as well as the distal arm (Figure 3D). The PMA analysis of 14 breast cancer cell lines and 20 tumor samples did not reveal promoter methylation in any of the samples.

### DEAR1 Restores Acinar Morphogenesis in 3D Basement Membrane Culture

**Figure 4:**
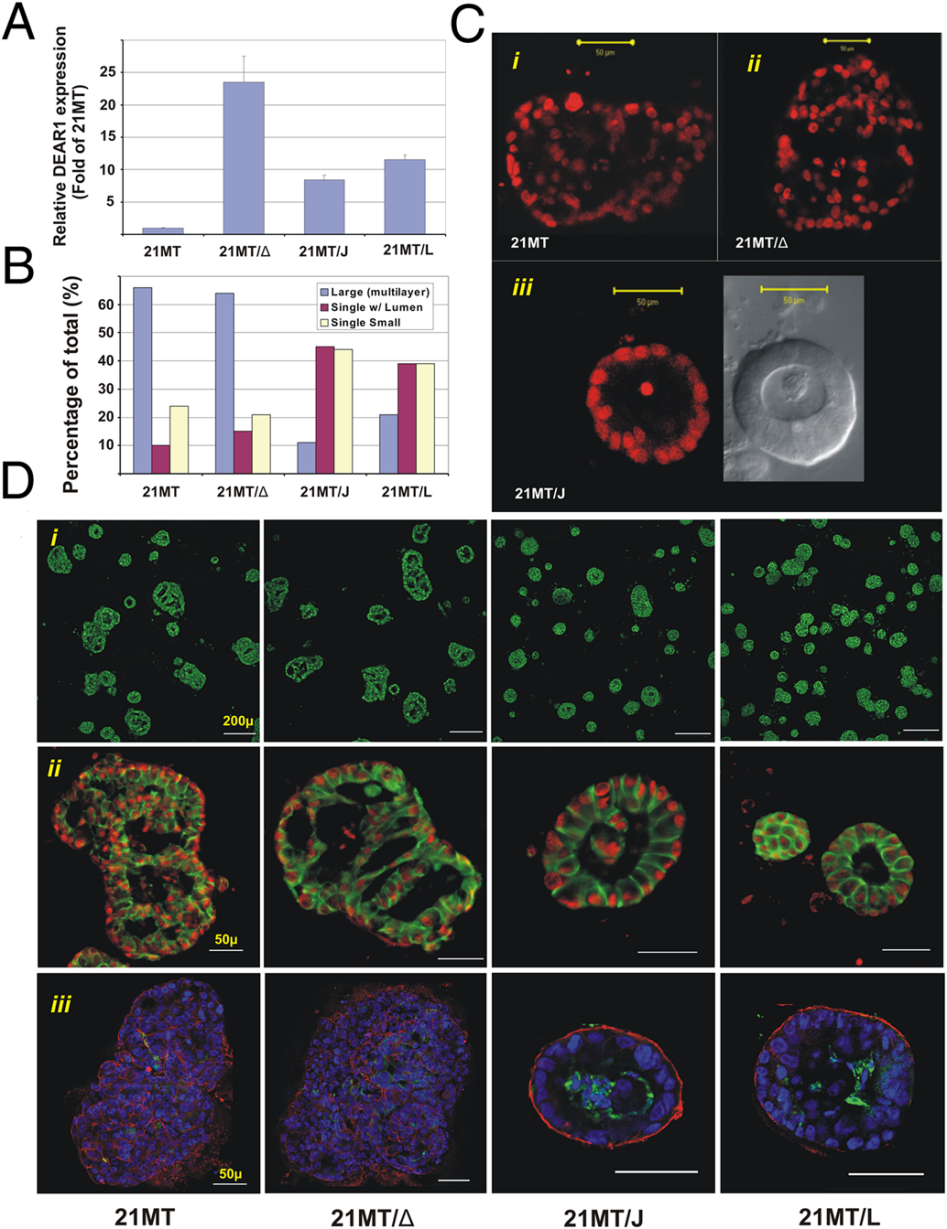
Introduction of DEAR1 mediates acinar morphogenesis in 3D culture. 21MT, control 21MT/Δ187, and wild-type transfectant 21MT/J and 21MT/L analyzed (A) by quantitative RT-PCR and (B) in 3D culture for the percentage of acinar structures. (C) Propidium (red)-staining structures were photographed by confocal microscopy after 11 d in 3D culture. The lumen can be clearly seen in the DIC photomicrograph to the right of the fluorescent image. (D) Confocal images of 21MT, 21MT/Δ, and wild-type transfectant 21MT/J and 21MT/L (*i*) at low magnification (bar = 200 µm) illustrating the dramatic size differences in acini from transfectants with and without wild-type *DEAR1* and compared with 21MT cells; (*ii*) after staining with propidium (red), and E-cadherin (green) discriminated the basal orientation of nuclei and expression of E-cadherin at cell–cell contacts in wild-type transfectant structures propagated in 3D culture as compared with the large, disorganized apolar structures in 21MT and 21MT/Δ cells (bar = 100 µm); (*iii*) introduction of wild-type *DEAR1* into 21MT cells resulted in acinar morphogenesis with epithelial cells surrounding a lumen illustrated by staining with propidium (blue), which denotes basal orientation of nuclei, basal orientation of alpha-6-integrin (red), and increased caspase 3 (green) staining in luminal structures in wild-type transfectants as opposed to 21MT and 21MT/Δ.

In order to determine if the mutations in *DEAR1* are important to the genesis or progression of breast cancer and are not mere “passenger” mutations, we performed functional assays. To determine the effect of genetic complementation of the missense mutation affecting codon 187 in the breast cancer progression model as well as in a breast tumor sample, full-length *DEAR1* wild type and R187W mutant cDNA were introduced into 21MT to generate stable transfectants. Quantitative RT-PCR confirmed up-regulation of DEAR1 RNA levels following stable transfection (Figure 4A). cDNA sequencing confirmed expression of predominant wild-type *DEAR1* transcripts in 21MT/J and 21MT/L transfectants and as well as the R187W mutant transcripts in control 21MT/Δ (unpublished data). Protein expression levels on Western analyses were very similar among transfectants and controls, including HMECs (Figures 2C and S4). 21MT cells, wild-type transfectants (21MT/J) and (21MT/L), and R187W transfectant 21MT/Δ were then plated in 3D basement membrane culture. Results indicated that over 60% of 21MT cells in 3D culture formed large, disorganized structures as determined by staining with propidium iodide followed by visualization using confocal microscopy (Figure 4B). Introduction of the tumor-associated R187W missense mutation in 21MT/Δ also resulted in a similar percentage of large, irregularly shaped multiacinar structures as observed in 21MT cells (Figure 4B). However, introduction of wild-type *DEAR1* into 21MT cells resulted in acinar morphogenesis with >80% of wild-type transfectants producing small, spherical acini. Forty percent of these structures contained a central lumen surrounded by a single layer of polarized epithelial cells (Figure 4B and 4D [*i*]) unlike the vast majority of multiacinar structures observed in 21MT and missense mutant controls as visualized by confocal and differential interference contrast (DIC) microscopy (Figure 4B, 4C, and 4D [*i*]). Thus, the morphological appearance of wild type transfectants was strikingly similar to normal acini formed by HMECs in 3D culture.

On day 9 of 3D culture, DEAR1 transfectants (*n* = 50) had a median diameter in 3D culture of 71.0 µm (interquartile range, 58.6 to 91.9 µm; range, 43.2 to 167.3 µm) for full-length DEAR1 transfectant J and 69.8 µm (interquartile range, 60.2 to 85.0 µm; range, 40.7 to 139.8 µm) for transfectant (L). The diameter of 21MT structures in 3D culture measured 108.6 µm (interquartile range, 81.3 to 166.6 µm; range, 48.8 to 394.1 µm; *n* = 50) which was significantly different from acini formed by transfectants with *DEAR1* (using a Mann-Whitney statistical analysis, *p*<0.0001). Similarly, transfectant 21MT/Δ containing the codon 187 missense mutation resulted in structures (median, 128.5 µm; interquartile range, 88.9 to 176.0 µm; range, 38.1 to 304.6 µm; *n* = 50), which by size and morphology closely resembled 21MT cells in basement membrane culture and were significantly different from *DEAR1* wild-type transfectants (*p*<0.0001). Staining with E-cadherin allowed the visualization of cell–cell contacts and emphasized the distorted cell structures in 21MT and 21MT/Δ in which cells of various sizes and shapes were observed with many misshapen cells visualized by confocal microscopy (Figure 4D [*ii*]). 21MT and 21MT/Δ transfectants in 3D culture also showed diminished polarized expression of alpha-6-integrin, which is normally expressed on the basolateral surface at the cell membrane (Figure 4D). In contrast to 21MT and 21MT/Δ, E-cadherin staining in wild-type *DEAR1* transfectants was properly localized at cell–cell contacts. Furthermore, acini displayed uniform cell size and clear basal orientation of nuclei with increased basal localization of alpha-6 integrin, indicating a restoration of ordered acinar architecture (Figure 4D [*iii*]). Furthermore, caspase 3 staining detected active luminal apoptosis in day 13 acinar structures in wild-type transfectant clones, recapitulating a defined event in normal mammary acinar morphogenesis (Figure 4D [*iii*]). In addition, results indicated no discernible difference in Ki67 staining in 3D cultures of 21MT versus wild-type or mutant transfectants at day 13 when wild-type transfectants were undergoing active luminal apoptosis (Figure S5), suggesting that DEAR1's influence on apoptotic rather than proliferative pathways is more evident in this model system. Thus, the introduction of wild-type *DEAR1* resulted in restoration of normal epithelial acinar architecture, a reinitiation of apicobasal polarity, and a clearing of luminal space, providing evidence for the role of *DEAR1* in the dominant regulation of acinar morphogenesis and indicating that the 21MT missense mutant phenotype could be rescued by the introduction of wild-type *DEAR1*. Similar results were obtained by transient transfection of *DEAR1* into MCF-7, which has very low to undetectable *DEAR1* expression (Figure 2C), in which transient expression of *DEAR1* could partially restore acinar morphogenesis in this cell line (Figure S6).

### Knockdown of *DEAR1* in Human Mammary Epithelial Cells Recapitulates the Phenotype of 21MT in 3D Culture

**Figure 5:**
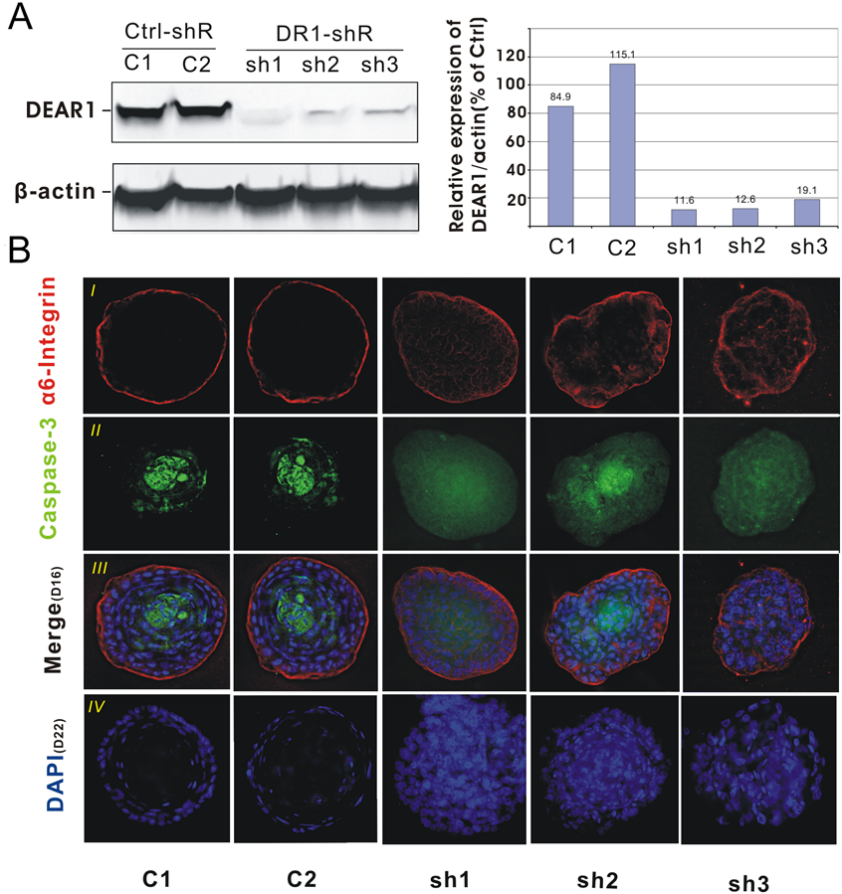
DEAR1 is a dominant regulator of acinar morphogenesis in HMECs. (A) Western analysis of shRNA control clones (C1 and C2) and shRNA knockdown clones (sh1, sh2, and sh3). (B) Confocal images of 3D cultures of control clones (C1 and C2) and *DEAR1*-knockdown clones (sh1, sh2, and sh3) showing representative acinus stained with alpha6-integrin (red), caspase 3 (green), or DAPI (blue), which shows the clear lumen in controls as opposed to shRNA knockdown clones (B *i, ii,* and *iii* are results at day 16; *iv* is at day 22).

To determine the effect of loss of function of DEAR1 in normal mammary differentiation, we silenced *DEAR1* expression in immortalized human mammary epithelial cells (76N-E6 cells) using lentiviral short hairpin RNA (shRNA). Three shDEAR1 clones as well as control shRNA clones were examined by Western analysis (Figure 5A) and for growth in 3D culture (Figure 5B). Results indicated that *DEAR1* stable knockdown clones (3/3), which were extensively silenced for *DEAR1* expression (Figure 5A), failed to form normal acini in 3D culture with irregular, asymmetric structures visible following 16 days in 3D culture (Figure 5B). Furthermore, cells within asymmetric structures appeared disorganized with ubiquitous staining for alpha-6 integrin, indicating loss of apical–basal polarity. Diffuse low to moderate staining for caspase 3 was also observed in shDEAR1 clones at day 16, during which time control HMECs demonstrated active luminal apoptosis. These results indicate that without *DEAR1*, apoptosis is not restricted to the lumen of acinar structures and moreover, three separate sh*DEAR1* clones failed to form lumens even after 22 d in culture as compared with control knockdown clones, which formed discrete lumens by the same time point (Figure 5B [*iv*]). In addition, BrdU incorporation in day 10 acinar structures indicated no apparent difference in proliferation between knockdown and control clones (Figure S5). Thus, stable silencing of *DEAR1* in immortalized, nontransformed human mammary epithelial cells disrupted normal acinar morphogenesis and recapitulated the phenotype observed in 21MT.

### Loss of DEAR1 Expression in Early Onset Breast Cancers Correlates with the Triple-Negative Phenotype of Breast Cancers with Poor Prognosis and Strong Family History of Breast Cancer

Because both *DEAR1* mutations and a homozygous deletion were observed in primary tumors from young women, and because we herein demonstrate the functional importance of complementation of a tumor-derived mutation and in vitro silencing of the gene, these data indicate that *DEAR1* is involved in the underlying genetic etiology of early-onset breast cancer. To address the clinical significance of DEAR1 in early-onset breast cancer, a well characterized tissue array from a cohort of 158 premenopausal women with onset of breast cancer between the ages of 25–49 years was screened by immunohistochemistry for *DEAR1* expression [39]. All of the tissue array samples were from stage I or II breast cancers treated with breast conservation surgery and postsurgical radiation therapy (Table 1). All progressed to invasive disease even though 72% of samples were from node-negative breast cancers. Interrogation of this array using the N-terminal DEAR1 antibody that we developed identified 56% of the tumor samples with complete loss of *DEAR1* expression, while 44% retained expression.

**Table 1:** Patient and tumor characteristics stratified by *DEAR1* expression.

Features	Number	*DEAR1* Expression		*p*-Value
		Negative	Positive	
**Histology**				0.6582
Ductal	100	55 (83%)	45 (88%)	
Lobular	5	2 (3%)	3 (6%)	
Others	12	9 (14%)	3 (6%)	
**Tumor size**				0.1463
T_1_	75	47 (75%)	28 (61%)	
T_2_	34	16 (25%)	18 (39%)	
**Nodal status**				1.0000
Negative	74	43 (73%)	31 (72%)	
Positive	28	16 (27%)	12 (28%)	
**ER**				0.4253
Negative	71	41 (68%)	30 (60%)	
Positive	39	19 (32%)	20 (40%)	
**PR**				0.0321
Negative	68	43 (70%)	25 (49%)	
Positive	44	18 (30%)	26 (51%)	
**HER-2**				0.7526
Negative	103	57 (92%)	46 (90%)	
Positive	10	5 (8%)	5 (10%)	
**Triple negative**				0.0362
No	58	26 (43%)	32 (64%)	
Yes	52	34 (57%)	18 (36%)	
**p53**				1.0000
Negative	85	46 (75%)	39 (76%)	
Positive	27	15 (25%)	12 (24%)	
**Strong family history**				0.0139
No	92	46 (73%)	46 (92%)	
Yes	21	17 (27%)	4 (8%)	
**BRCA1 mutation**				0.6347
No	47	28 (88%)	19 (95%)	
Yes	5	4 (12%)	1 (5%)	
**BRCA2 mutation**				0.5173
No	50	30 (94%)	20 (100%)	
Yes	2	2 (6%)	0 (0%)	

Clinical parameters for the cohort under study were analyzed for statistical significance with *DEAR1* expression. The analysis included two groups: samples scored as either focal or diffusely positive in the positive group and all samples scored as total absence of staining in the negative group. Thirty-five of the 158 total samples were not scorable due to loss of tissue. Results on 123 samples indicated that *DEAR1* loss of expression did not correlate significantly with tumor size (correlation coefficient: *r* = 0.15), lymph node metastasis (*r* = 0.01), race (*r* = −0.03), ER (*r* = 0.09), HER-2 (*r* = 0.03), or p53 (*r* = −0.01) expression status (Table 1). *DEAR1* loss of expression did not correlate with BRCA1 or BRCA2 mutation, but rather, loss of expression correlated with a strong family history of breast cancer in this young cohort (*r* = −0.24, *p* = 0.0139). Seventeen of 21 individuals represented on the tissue array with a strong history of breast cancer in their families were negative for DEAR1 staining. Furthermore, loss of *DEAR1* expression correlated significantly with loss of progesterone receptor expression and with the triple-negative phenotype (ER^−^, PR^−^, HER-2^−^) of breast cancers (*r* = 0.21, *p* = 0.0362), a subgroup common in BRCA1 mutation carriers and identified by gene expression profiling as breast cancers of poor prognosis and for which few treatment options exist (Table 1) [40]. Together, the loss of expression of *DEAR1* in the majority of early-onset cases examined and its correlation with family history and the triple-negative phenotype strongly supported further investigation of this gene as a candidate biomarker in early-onset breast tumors.

### *DEAR1* Expression is an Independent Predictor of Local Recurrence-Free Survival in Early Onset Breast Cancer

**Figure 6:**
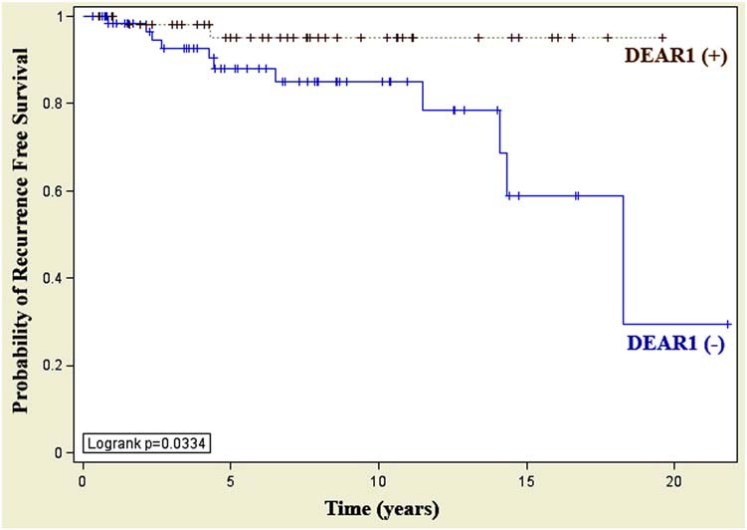
DEAR1 is an independent predictor of local recurrence free survival in early onset breast cancer. Immunohistochemical staining of an early onset tissue array resulted in a significant correlation between the expression of DEAR1 and the probability of local recurrence free survival (*p* = 0.0334). At 15 y post diagnosis, recurrence-free survival in *DEAR1*-negative patients was 58% compared to 95% in those patients whose tumors were positive for *DEAR1* expression.

Although loss of *DEAR1* expression did not correlate with distant metastasis or survival in this young cohort of women with early stage breast cancer, loss of *DEAR1* expression on immunohistochemical staining significantly predicted local recurrence. At 5-y follow-up, DEAR1-positive expression correlated significantly, with a 95% local recurrence-free survival and this survival rate did not change in our cohort for over 15 y postsurgical follow-up. In contrast, for samples demonstrating loss of expression of *DEAR1*, recurrence-free survival fell to 80% at 10 y and 58% at 15 y (*p* = 0.034) (Figure 6). Thus, these data indicate that *DEAR1* expression is an independent predictor of local recurrence in early-onset breast cancers and suggest that DEAR1-negative staining on immunohistochemistry could be an important marker to stratify early-onset breast cancer patients for increased vigilance in follow-up and adjuvant therapy.

## Discussion

Herein we describe the identification of the novel gene *DEAR1* and provide evidence for its role in the dominant regulation of acinar morphogenesis in three-dimensional culture. *DEAR1* undergoes mutation and deletion in breast cancer. Furthermore, by complementation of a somatic tumor-derived missense mutation, wild-type *DEAR1* restored acinar structures that, by size, polarity, and presence of luminal apoptosis, resembled normal mammary acini grown under similar conditions. Stable knockdown of *DEAR1* in immortalized HMECs recapitulated the phenotype in 21MT cells with disruption of tissue architecture, loss of polarity, and lumen formation, indicating that *DEAR1* is required for normal acinar morphogenesis in 3D culture. Together, these data define *DEAR1* as a critical link between the control of tissue architecture via ECM remodeling and a tumor-specific mutational event in breast cancer.

*DEAR1* is also a new member of the RBCC/TRIM family of RING finger proteins, which have been intimately associated with development, differentiation, and oncogenesis. Although RBCC/TRIM family members are functionally diverse, these proteins are considered critical regulators of the cellular architecture of large protein complexes [29]–[32]. To date, mutations in RBCC/TRIM family members have been shown to be causal in hereditary disorders of development, including mutation of *MUL* in mulibrey nanism, an autosomal recessive disorder involving defective development of several mesodermal tissues and *MID1*, in X-linked Opitz/GBBB syndrome, an inherited disorder primarily affecting midline structures as well as PYRIN/MARENOSTRIN, which is specifically mutated in familial Mediterranean fever [41]–[45]. The tumor suppressor *PML* is the only RBCC/TRIM family member of which we are aware for which cancer-specific mutations have been observed [46]. *DEAR1,* thus, represents the second example of an RBCC/TRIM family member that is specifically mutated in cancer and the only family member for which functional studies link loss of differentiation to a cancer-specific mutation.

A number of published reports have elucidated the critical role of microenvironmental signaling in the maintenance of epithelial cell differentiation. Elegant studies using 3D culture have allowed the experimental targeted manipulation of key signaling pathways that dramatically altered the differentiation state of invasive tumor cells to one resembling a more normal cell phenotype irrespective of the genetic alterations in the tumor cell genome. Thus, although it is experimentally feasible to phenotypically alter the ECM and the growth of tumor cells in vivo and in vitro, we now have genotypically complemented a tumor-associated mutation, indicating that replacement of a single gene can restore epithelial differentiation despite multiple genetic abnormalities in a breast cancer cell line and, furthermore, that *DEAR1* is a dominant regulator of an important pathway to tumorigenesis in early-onset breast cancer.

In that regard, critical pathways underlying transformation and malignant progression in mammary tumorigenesis involve a disruption of normal controls on proliferation, on epithelial architecture and polarity [7]–[9]. In mammalian cells, mechanisms governing polarity and proliferation have been shown to involve separate pathways. Phosphatidylinositol 3-kinase (PI3K) signaling through AKT drives proliferation in mammary acini, whereas PI3K–RAC signaling is necessary for loss of tissue organization [47]. The oncogene ErbB2, overexpressed in 25%–30% of breast cancer, has been shown to disrupt polarity by associating with the Par6 polarity complex [48]. Integrin β4–ErbB2 association has also been shown to disrupt polarity and growth control by separate mechanisms involving activation of STAT3, controlling polarity and c-Jun, resulting in proliferation [49]. In addition, loss of function of the polarity protein Scribble cooperates with c-myc to drive mammary tumorigenesis [50]. Our results in 3D culture suggest that there is no discernible difference in Ki67 staining of acini in the *DEAR1* knockdown HMECs compared with wild-type HMECs, suggesting that *DEAR1* mediates its effects more by regulation of polarity than by proliferation, although additional experimentation will be required to dissect the pathways regulated by DEAR1. DEAR1's role in regulating polarity and its loss of function in breast cancer provides an intriguing glimpse into a novel regulatory circuitry that goes awry in early onset breast cancer.

*DEAR1* maps within one of the most frequent regions of LOH in breast cancers with poor prognosis in node negative breast cancers as well as a genomic interval associated with LOH in many histologically diverse epithelial cancers, suggesting that it could be a candidate tumor suppressor in the region [25]–[28]. Also of importance is the recent finding of Bagchi et al. in which chromosome engineering identified *CHD5* as a candidate tumor suppressor within Chromosome 1p36, distal to *DEAR1* at 1p35.1 [51]. *CHD5* maps to a region associated with LOH in epithelial tumors, as well as brain tumors and hematopoietic neoplasms, suggesting that *CHD5* is a critical player in many types of cancer. Interestingly, in the breast tumor sample showing HD for *DEAR1*, one copy of Chromosome 1p contains a microdeletion of *DEAR1*, while the second copy deletes the entire short arm, including *CHD5*, but the breakpoint for the deletion lies within *DEAR1*. *CHD5* maps to a genomic interval associated with LOH in late-stage tumors; thus, the finding that *DEAR1* seems to play a role in the earliest stages of breast tumorigenesis would suggest a mechanism for mutation or deletion of *DEAR1* as an initiating event that could lead to the LOH for distal Chromosome 1p loci and thus haploinsufficiency of *CHD5*.

The present study also describes the potential clinical significance of *DEAR1* genetic alteration and loss of expression in breast tumorigenesis. Mutation and homozygous deletion of *DEAR1* were discovered in young women. We therefore examined the 21T series as a model to determine if *DEAR1* could be functionally linked to early onset disease. Our data indicate that *DEAR1* mutation and loss of function play a role in early-onset disease. These data do not indicate, however, that *DEAR1* does not play a role in breast cancer in older women or that DEAR1 discriminates breast cancers by age. Rather, these data highlight that *DEAR1* plays a role in the etiology of breast cancer in young women. Intriguingly, *DEAR1* maps to a genomic interval for which both linkage and LOH in familial breast cancers have been reported [26]. Our data demonstrate that in our young cohort, DEAR1 correlates significantly with triple-negative cancers as well as a strong family history of breast cancer. Thus, it is a formal possibility that *DEAR1* loss of function and mutation might play an important role in germline predisposition to breast cancer or that *DEAR1* lies in a critical genetic pathway involved in both inherited and sporadic breast cancer. The loss of function of upstream pathway members could inactivate *DEAR1* expression, a potential explanation for the higher frequency of loss of expression than mutation that we observed.

*DEAR1* expression was also a statistically significant prognostic marker for local recurrence-free survival over 20 y postsurgery. Previously, this cohort had been examined for markers that might predict local recurrence, including ER, PR, HER-2/neu, p53, and cytokeratin 19; however, only cytokeratin 19 was statistically significant for predicting local recurrence [39]. The finding that *DEAR1* expression independently predicts local recurrence in early onset disease is important given that local recurrence following breast conservation surgery in younger women is a major clinical issue. Young women with breast cancer have significantly higher rates of local recurrence than older women, with local recurrence following breast conservation therapy and radiotherapy occurring earlier and with a worse prognosis in many studies than in older cohorts [52]–[55].

Thus, there is an urgent need to identify prognostic markers to identify women with a heightened risk of recurrence for which more aggressive surveillance and treatment might be warranted, as well as individuals with favorable prognosis who might be spared rigorous therapeutic regimens and for whom breast conservation treatment might be the preferred surgical option. Our data suggest that *DEAR1* loss of function may play an important role in the loss of differentiation and the poor outcome associated with a high frequency of early-onset cancers. The finding that *DEAR1* correlates with the triple-negative breast cancer subtype also suggests an impact of loss of *DEAR1* expression on the differentiated state in this subtype of basal tumors of the breast. Thus, the clear delineation between *DEAR1* expression and recurrence, and the correlation of *DEAR1* expression with the subtype of breast tumors with poor prognosis, suggest that *DEAR1* is an important biomarker for stratifying early-onset disease; and these data in conjunction with its role as a dominant mediator of differentiation in 3D culture point to a critical role for *DEAR1* in a genetic pathway that is important in early-onset breast cancer, the elucidation of which could have an important impact on early detection and targeted therapy for malignancies of the breast.

## Supporting Information

Figure S1 Suppression subtractive hybridization cloning of *DEAR1*. Microcell hybrids were constructed by the introduction of a normal copy of Chromosome 3 or fragments of Chromosome 3p into a renal cell carcinoma (RCC) cell background [18]–[21],[56]. Microcell hybrids were injected subcutaneously or orthotopically in athymic nude mice. Results indicated that the entire Chromosome 3 suppressed the formation of tumors and that a small centric fragment (3p12-q11) also suppressed tumors; however, a fragment containing a deletion in the 3p12 region (3p12-q24) failed to suppress tumors, mapping a functional tumor suppressor locus to a 4.75 Mb interval within chromosome 3p12. Microcell hybrids were used as starting materials for SSH library construction. *DEAR1* was isolated as one of the cDNAs present in the SSH library.
(9.98 MB TIF)

Figure S2 FISH mapping of *DEAR1*. (A) Chromosomal localization of *DEAR1* as observed by FISH analysis using the *DEAR1* P1-derived artificial chromosome (PAC) clone. Strong signal was observed in the distal region of Chromosome 1p. Based on physical mapping, *DEAR1* was mapped to the 1p35.1 interval. (B) The 420 kb region harboring *DEAR1* is shown in the center of the figure with flanking genes identified. As denoted by the bracket on the Chromosome 1 ideogram, the 1p34-35 region has been shown to have high frequency LOH in sporadic breast cancers with poor prognosis as well as familial breast cancers.
(0.18 MB TIF)

Figure S3 DEAR1 is a highly evolutionarily conserved protein. Alignment of the human, mouse, and rat *DEAR1* protein sequences demonstrates significant similarity. Amino acid identity is denoted by “*” in the consensus line, a conserved substitution is denoted by “:”, and a non-conserved substitution is indicated with a blank space.
(2.49 MB TIF)

Figure S4 Effect of cycloheximide on DEAR1 protein levels in the 21MT series cell lines. Lysates from 21MT, 21MT/Δ, 21MT/J, and 21MT/L cells treated with 50 µg/ml cycloheximide were analyzed by immunoblotting. The p21 control shows loss of stability following the same treatment.
(3.24 MB TIF)

Figure S5 Effect of DEAR1 on cell proliferation markers in 3D culture. Top panel: Ki-67 expression in 21MT series. Bottom panel: BrdU incorporation in DEAR1-KD clones and control clones.
(9.84 MB TIF)

Figure S6 Effect of DEAR1 on restoring acinar morphogenesis in MCF-7 cells in 3D culture. (A) DEAR1 expression was detected from cell lysates on Western blots after *DEAR1* transient transfection into MCF7. (B) Acinar morphogenesis of MCF7 cells transiently expressing *DEAR1* compared with vector at day 19.
(9.68 MB TIF)

Text S1 Experiments and methods.
(0.05 MB DOC)

Table S1 *DEAR1* genetic alterations in breast cell lines.
(0.02 MB DOC)

Table S2 *DEAR1* genetic alterations in breast tumors.
(0.03 MB DOC)

Table S3 Primers used to identify a homozygous deletion in breast tumors.
(0.04 MB DOC)

## Abbreviations

DCIS: ductal carcinoma in situ
DHPLC: denaturing high-performance liquid chromatography
DIC: differential interference contrast
ECM: extracellular matrix
FISH: fluorescence in situ hybridization
HD: homozygous deletion
HMEC: human mammary epithelial cell
LOH: loss of heterozygosity
PMA: pyrosequencing-based methylation analysis
TRIM: tripartite motif
