# Supplementary figures and images for: DEAR1 Is a Dominant Regulator of Acinar Morphogenesis and an Independent Predictor of Local Recurrence-Free Survival in Early-Onset Breast Cancer
Source: PLoS Med. 2009 May 5;6(5):e1000068. doi: 10.1371/journal.pmed.1000068 (PMC2673042; doi:10.1371/journal.pmed.1000068)

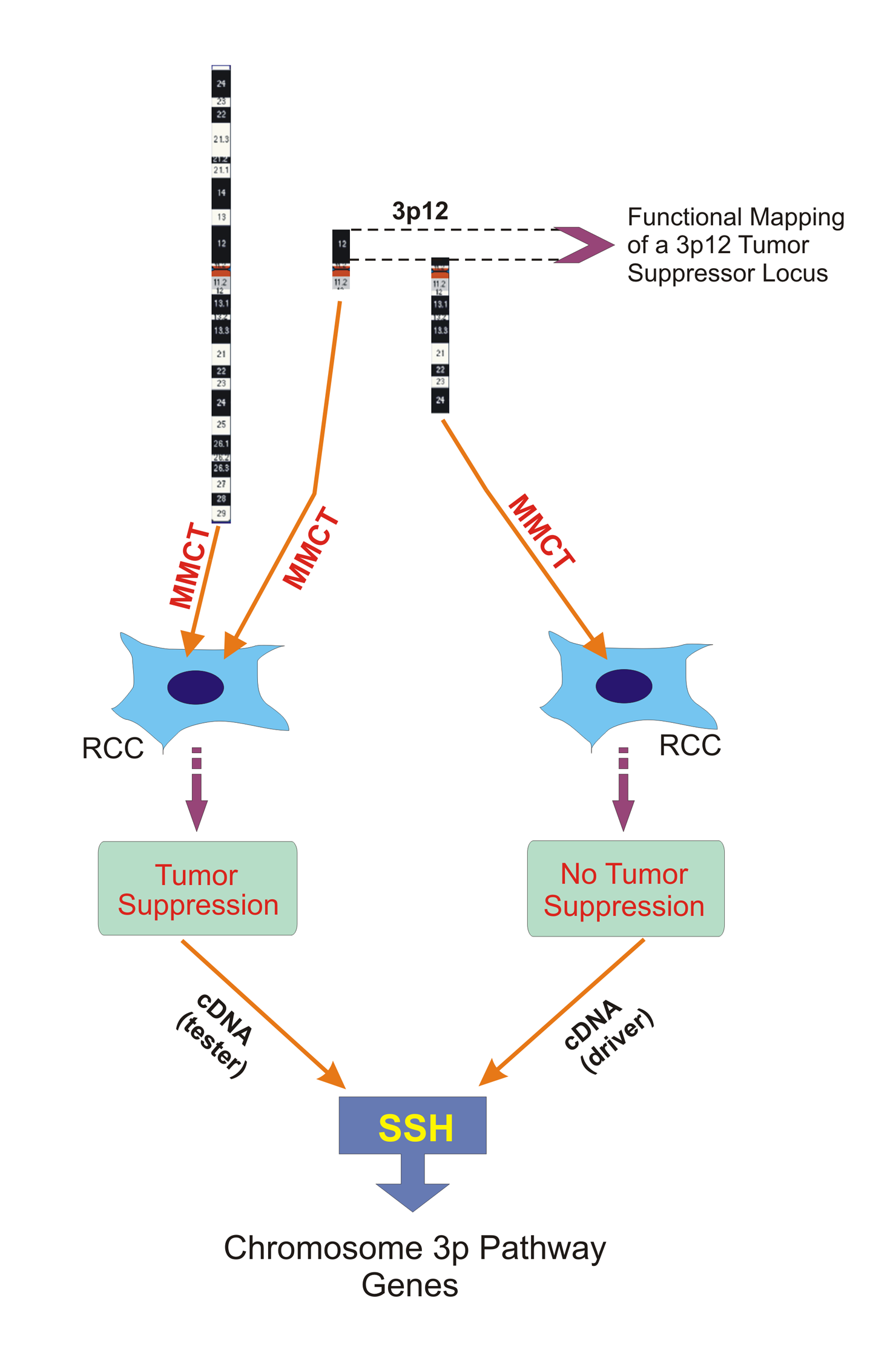

Supplement: Figure S1 — Suppression subtractive hybridization cloning of DEAR1. Microcell hybrids were constructed by the introduction of a normal copy of Chromosome 3 or fragments of Chromosome 3p into a renal cell carcinoma (RCC) cell background [18]–[21],[56]. Microcell hybrids were injected subcutaneously or orthotopically in athymic nude mice. Results indicated that the entire Chromosome 3 suppressed the formation of tumors and that a small centric fragment (3p12-q11) also suppressed tumors; however, a fragment containing a deletion in the 3p12 region (3p12-q24) failed to suppress tumors, mapping a functional tumor suppressor locus to a 4.75 Mb interval within chromosome 3p12. Microcell hybrids were used as starting materials for SSH library construction. DEAR1 was isolated as one of the cDNAs present in the SSH library. (9.98 MB TIF) [file pmed.1000068.s001.tif]

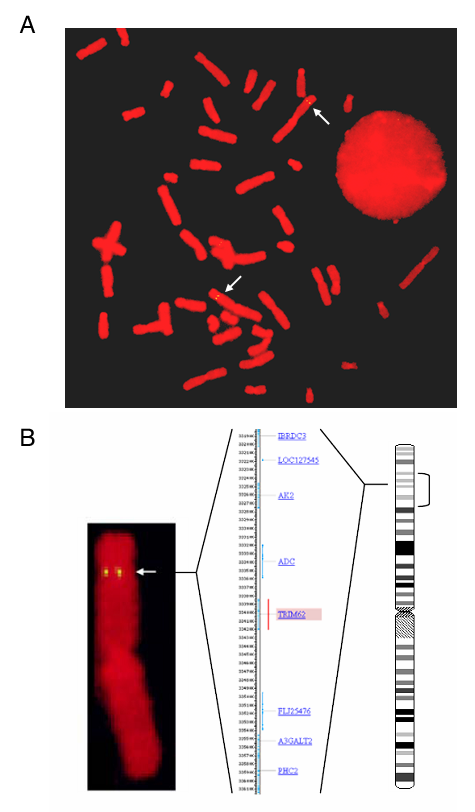

Supplement: Figure S2 — FISH mapping of DEAR1. (A) Chromosomal localization of DEAR1 as observed by FISH analysis using the DEAR1 P1-derived artificial chromosome (PAC) clone. Strong signal was observed in the distal region of Chromosome 1p. Based on physical mapping, DEAR1 was mapped to the 1p35.1 interval. (B) The 420 kb region harboring DEAR1 is shown in the center of the figure with flanking genes identified. As denoted by the bracket on the Chromosome 1 ideogram, the 1p34-35 region has been shown to have high frequency LOH in sporadic breast cancers with poor prognosis as well as familial breast cancers. (0.18 MB TIF) [file pmed.1000068.s002.tif]

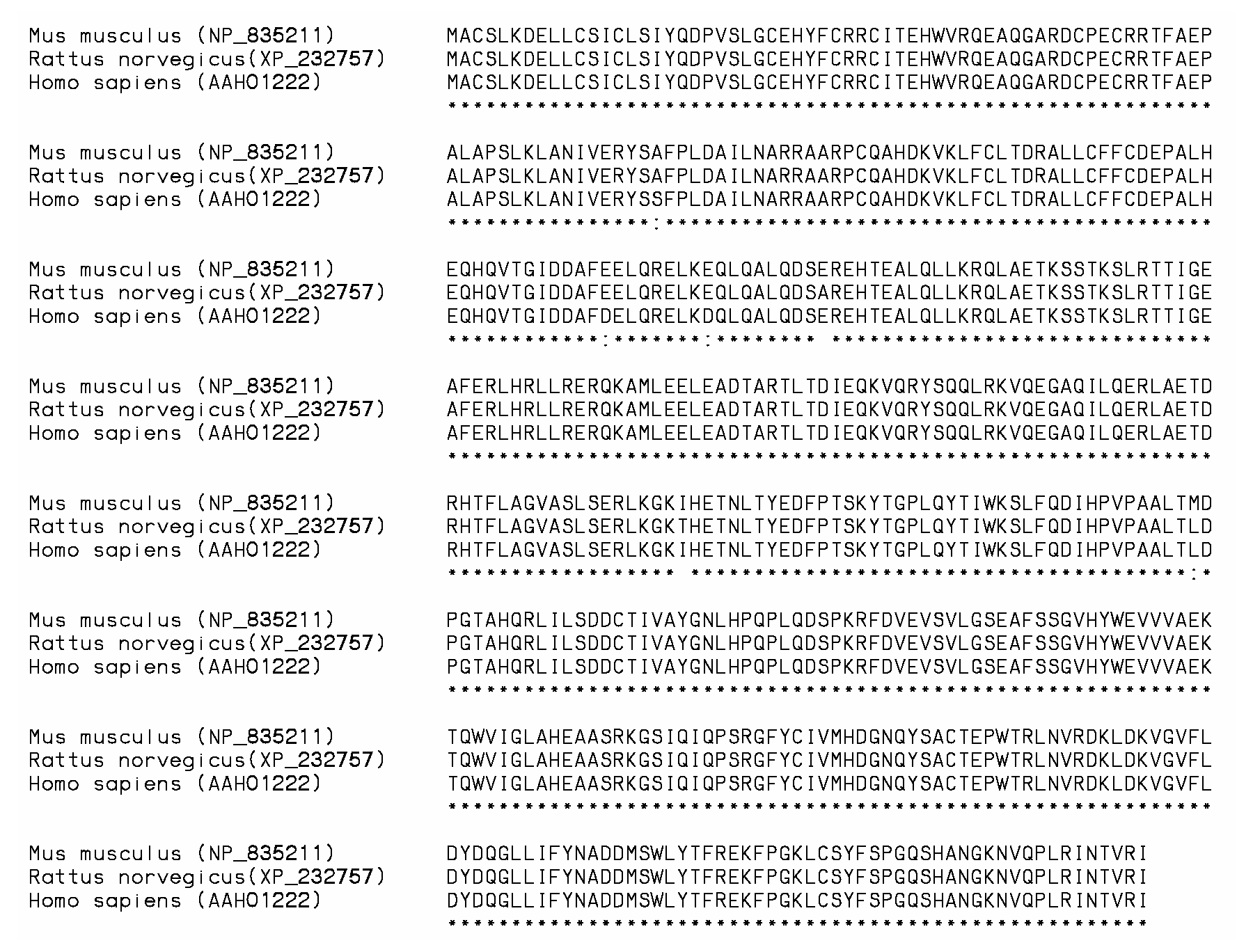

Supplement: Figure S3 — DEAR1 is a highly evolutionarily conserved protein. Alignment of the human, mouse, and rat DEAR1 protein sequences demonstrates significant similarity. Amino acid identity is denoted by “*” in the consensus line, a conserved substitution is denoted by “:”, and a non-conserved substitution is indicated with a blank space. (2.49 MB TIF) [file pmed.1000068.s003.tif]

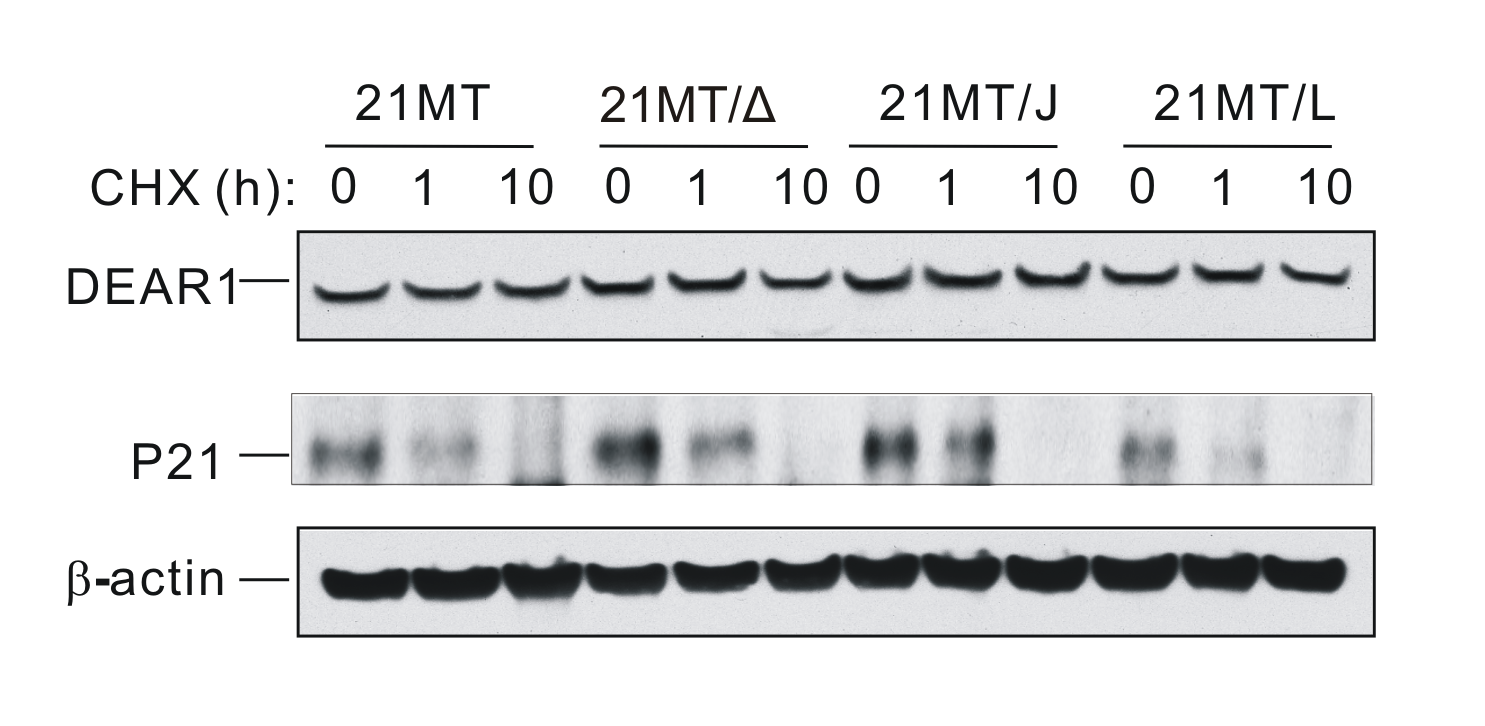

Supplement: Figure S4 — Effect of cycloheximide on DEAR1 protein levels in the 21MT series cell lines. Lysates from 21MT, 21MT/Δ, 21MT/J, and 21MT/L cells treated with 50 µg/ml cycloheximide were analyzed by immunoblotting. The p21 control shows loss of stability following the same treatment. (3.24 MB TIF) [file pmed.1000068.s004.tif]

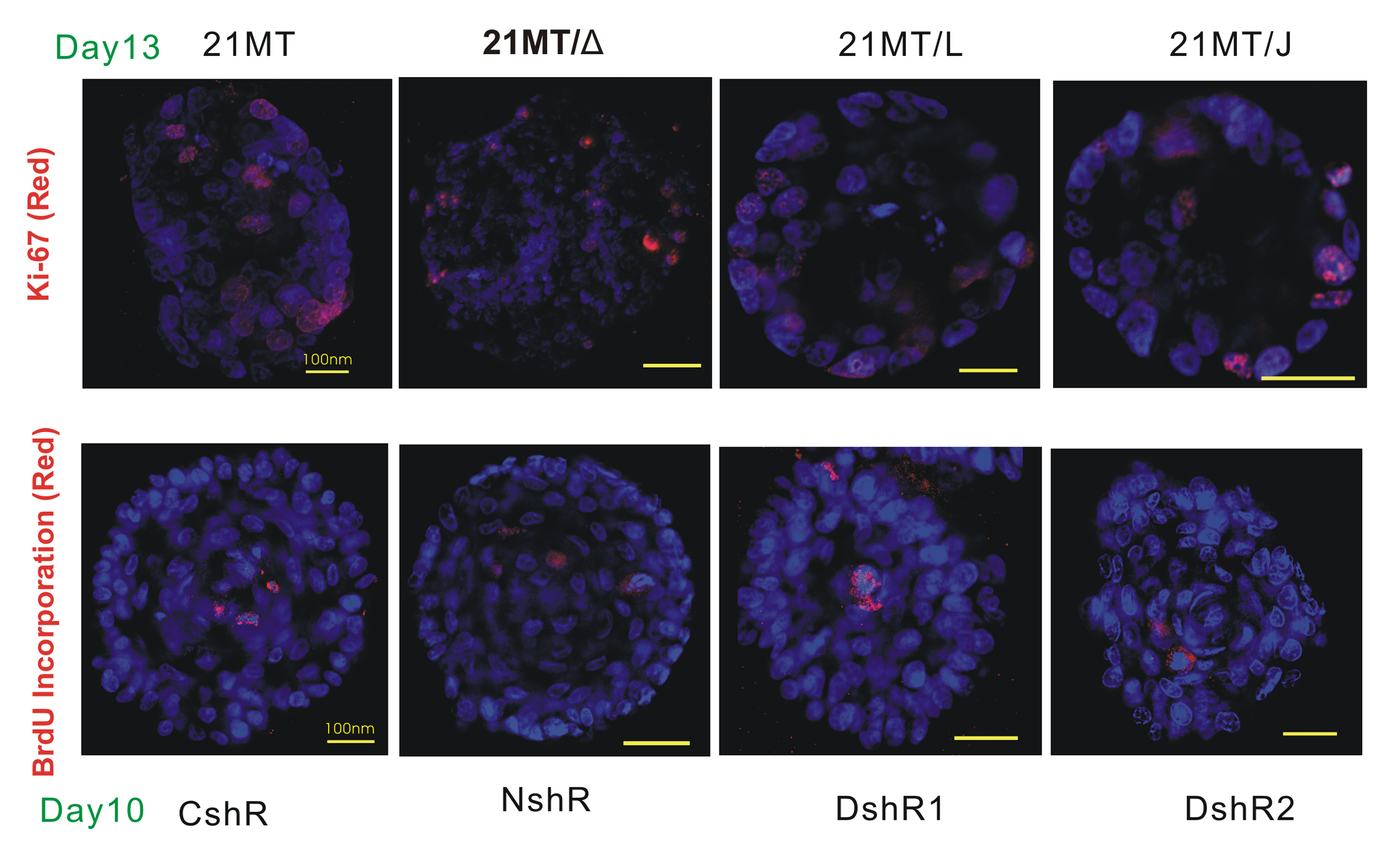

Supplement: Figure S5 — Effect of DEAR1 on cell proliferation markers in 3D culture. Top panel: Ki-67 expression in 21MT series. Bottom panel: BrdU incorporation in DEAR1-KD clones and control clones. (9.84 MB TIF) [file pmed.1000068.s005.tif]

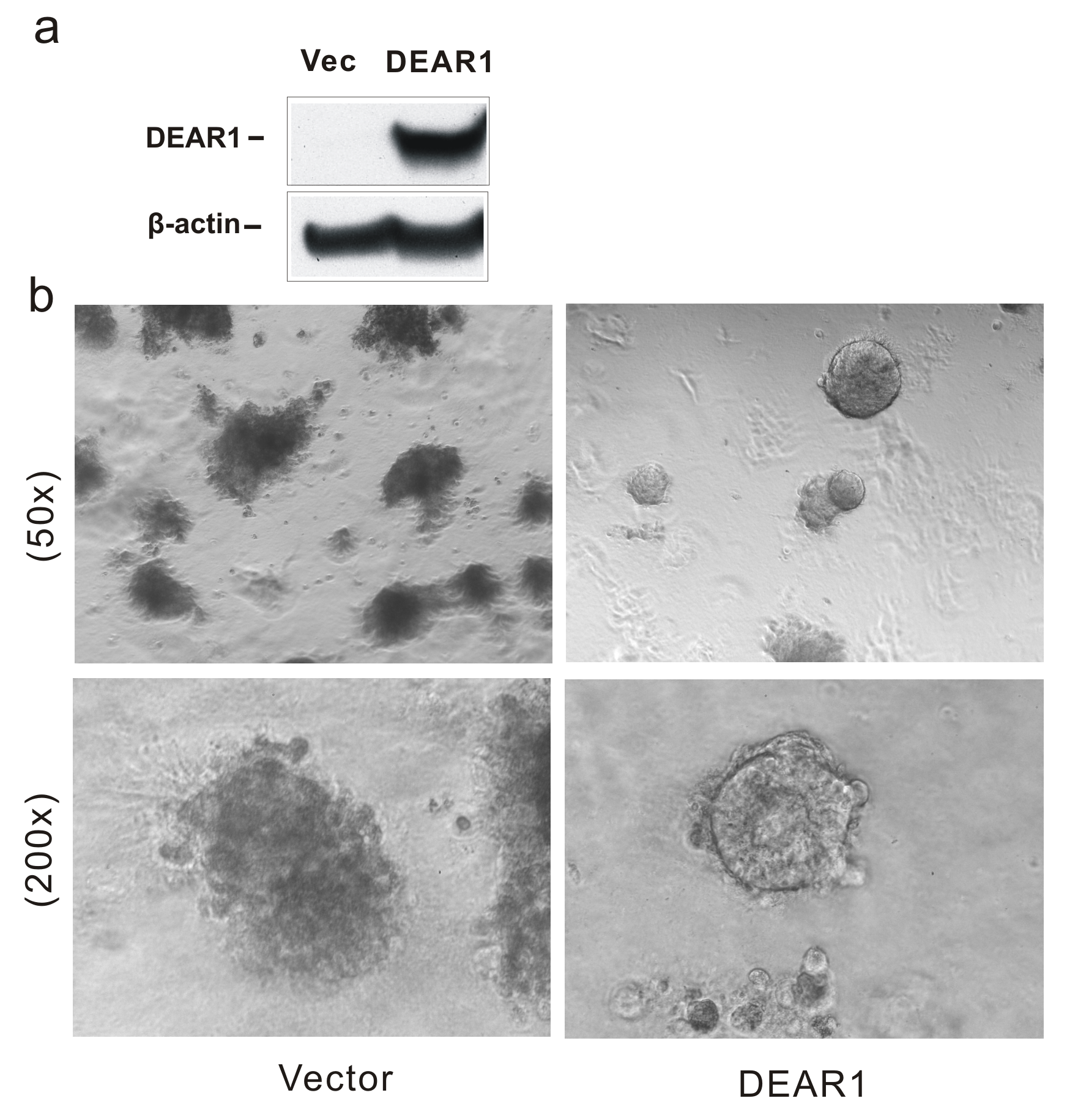

Supplement: Figure S6 — Effect of DEAR1 on restoring acinar morphogenesis in MCF-7 cells in 3D culture. (A) DEAR1 expression was detected from cell lysates on Western blots after DEAR1 transient transfection into MCF7. (B) Acinar morphogenesis of MCF7 cells transiently expressing DEAR1 compared with vector at day 19. (9.68 MB TIF) [file pmed.1000068.s006.tif]
