# Supplementary material for: DEAR1 Is a Dominant Regulator of Acinar Morphogenesis and an Independent Predictor of Local Recurrence-Free Survival in Early-Onset Breast Cancer
Source: PLoS Med. 2009 May 5;6(5):e1000068. doi: 10.1371/journal.pmed.1000068 (PMC2673042; doi:10.1371/journal.pmed.1000068)
Supplement: Text S1 — Experiments and methods. (0.05 MB DOC) [file pmed.1000068.s007.doc]

# Supporting Information Text S1

***DEAR1* is a Dominant Regulator of Acinar Morphogenesis and an Independent Predictor of Local Recurrence-Free Survival in Early Onset Breast Cancer**

**S.T. Lott et. al.**

# 1. A Functional Screen Identifies *DEAR1* in a Cytogenetic Pathway to Tumorigenesis.

# The single most common structural chromosome deletion in breast cancer is loss of chromosome 3p12-14, and in many cases loss of 3p is the sole cytogenetic aberration, suggesting deletion of this genomic region is a very early event in breast tumorigenesis [1-9]. We previously used a functional approach to map a novel tumor suppressor locus within chromosome 3p12 using microcell hybrid clones generated by the transfer of normal fragments of chromosome 3p into a renal cell carcinoma cell line which were subsequently tested for functional tumor suppressor activity following injection into athymic nude mice. Using this approach, we mapped a novel tumor suppressor locus within a 4.75 Mb interval of chromosome 3p12 [10-14].

# We then generated a suppression subtractive hybridization library (SSH) using as starting materials microcell hybrid clones containing defined fragments of chromosome 3p which were either suppressed (SN19(3i)YY [tester]) or unsuppressed for tumorigencity (SN19(3)EEE [driver]) to identify cDNAs that were differentially expressed and could represent a functional chromosome 3p pathway to tumorigenesis (Fig. S1).

**2. Suppression Subtractive Hybridization Cloning of DEAR1.** Tester SN19(3i)YY cDNA (suppressed hybrid clone) was hybridized to excess of "driver" cDNA derived from the unsuppressed and highly malignant hybrid line SN19(3)EEE. The resultant unhybridized cDNA was obtained representative of either genes differentially expressed in the region of nonoverlap within 3p12, or genes that are activated as a result of the introduction of the *NRC-1* locus supplied by the introduced chromosome 3p12 fragment (Figure. S1). As a first step in the protocol, cDNA was synthesized from 0.5-2 µg poly(A)RNA from SN19(3i)YY and SN19(3i)EEE. Tester and driver cDNAs are digested with RsaI, a four-base-cutting restriction enzyme that yields blunt ends. The tester cDNA was then subdivided into two portions and each was ligated with a different cDNA adapter. The ends of adapters lack phosphate groups, so only one strand of each adapter attaches to the 5' ends of the cDNA. The two adapters differ in sequence such that annealing of two different PCR primers is possible once the recessed ends have been filled in. The first hybridization reaction was performed in which the concentration of high and low abundance sequence was equalized among ss tester cDNAs since the more abundant molecules reanneal more rapidly by second order hybridization kinetics. For this step, an excess of driver was added to each sample of tester (driver cDNA has no adapters). The samples were then heat denatured and allowed to anneal. Also, the first hybridization essentially subtracts out common non-target tester cDNAs that form hybrids with driver cDNA. In the second hybridization, the fraction that corresponds to differentially expressed mRNAs will form viable templates for exponential amplification. Using suppression PCR, only differentially expressed sequences are amplified exponentially. Suppression occurs when complementary sequences are present on each end of a single-stranded cDNA. During each primer-annealing step, kinetics of hybridization favor pan-like structure formation that prevents primer annealing. When a primer anneals and is extended, the newly synthesized strand will also have the inverted terminal repeats and form another pan-like structure. During PCR, then, nonspecific amplification is suppressed, and PCR amplification of specific cDNA molecules with different adapters occurs.

The entire population was subjected to PCR to amplify specifically differentially expressed sequences. During this PCR step, only those molecules with two different annealing sites can be amplified exponentially. These should be the differentially expressed sequences. A second PCR was then performed to enrich for differentially expressed sequences and reduce any background PCR products. Differentially expressed cDNAs were inserted into a TA-cloning vector using the RsaI site at the adapter/cDNA junction and used to transform competent E. coli.

From this screen, a collection of clones containing partial cDNA inserts of sizes ranging from 150-1100 bp were obtained. Screening of cDNAs using a monochromosomal microcell hybrid panel identified a cDNA, *DEAR1,* that mapped to chromosome 1 and by fluorescence in situ hybridization (FISH) to a cytogenetic interval within chromosome 1p34-35 that has been shown to be deleted downstream of chromosome 3p loss in a cytogenetic pathway model for lung cancer [15]. (Figure. S2) Initial BLAST results demonstrated DEAR1 to have significant similarity to a novel RING finger protein (3.9 x 10^-18^) and Northern studies further indicated that *DEAR1* expression was lost or downregulated in cell lines associated with chromosome 1p LOH (data not shown). We therefore hypothesized that *DEAR1* was a downstream pathway member in the chromosome 3p pathway to tumorigenesis.

##### 3. The *DEAR1* Sequence is Highly Conserved in Mouse and Rat. Sequence analysis indicated that DEAR1 represents a novel member of the RBCC/TRIM family member of RING finger proteins. *DEAR1* is essentially identical (98%) to mouse and rat sequences (NP_835211 [Mus musculus] and XP_232757 [Rattus norvegicus]) (Figure. S3).

**4. Stability of Mutant DEAR1 Protein.** To investigate if the R187W mutation observed in the 21T series cell lines as well as a primary breast tumor (both from young women) affected DEAR1 protein stability, we examined the half-life of DEAR1 by performing a cycloheximide chase experiment in which we exposed 21MT, 21MT/J, 21MT/L and 21MT/Δ to cycloheximide (50 μg/ml) for various times (0.5, 1, 1.5, 2, 4, 6, 8, and 10 h). Results indicate that DEAR1 is a stable protein over the indicated time intervals in cells with either the mutant or wild type allele, indicative that the R187W mutation does not affect DEAR1 stability (Figure S4).

**5. Ki67 and BrdU Staining of Acinar Structures in 3D Culture.** To address the role of DEAR1 in regulating proliferation during acinar morphogenesis, proliferation in acinar structures was examined in wild type and mutant transfectants and controls. Results indicated that there was no significant difference in Ki67 staining in 3D cultures of the 21MT versus wild type transfectants at day 13 when wild type transfectants are undergoing active luminal apoptosis (Figure S5). In addition, we also determined BrdU incorporation into *DEAR1* HMEC knockdown clones and controls in 3D culture at day 10. Thus, in the knockdown model as in the 21MT model, we did not observe a significant difference in Ki67 or BrdU staining at the time points indicated, suggesting that in this model system apoptotic, but not proliferation pathways are mainly affected by *DEAR1* (Figure S5).

**6. Effect of DEAR1 in Restoring Acinar Morphogenesis in MCF-7 Cells in 3D Culture**. To determine if DEAR1 could rescue a loss of function phenotype, we performed transient transfection experiments into MCF-7 cells which have very low to undetectable DEAR1 expression on Western analysis (Fig. 1c). Subsequent Western analysis confirmed DEAR1 upregulated expression in MCF-7 cells post transfection of pCMV-DEAR1 (Fig. S6a). Results from growth in 3D culture indicate that MCF-7 cells grow similarly to 21MT in that by day 19, they have a very irregular growth pattern with lack of normal acinar structures (Fig. S6b). However, introduction of DEAR1 resulted in an increase in more uniform and polar acinar structures, some of which had discrete lumen (Fig. S6). Thus, even in transient assay we were able to document that DEAR1 could partially revert aberrant MCF-7 growth in 3D culture.

1. **For Supporting Figures and Tables, refer to main article file.**

## Supporting References

## Teixeira, M.R., Pandis, N., Sverre, H. Cytogenetic clues to breast carcinogenesis. Genes, Chromosomes & Cancer.2002; 33:1-16

## Naylor SL, Johnson BE, Minna JD, and Sakaguchi AY. Loss of heterozygosity of chromosome 3p markers in small-cell lung cancer. Nature 1987; 329:451-454.

## Zbar B, Brauch H, Talmadege C, and Linehan M. Loss of alleles of luci on the short arm of chromosome 3 in renal cell carcinoma. Nature 1987; 327:721-724.

## Brauch H, Johnson B, Hovis J, et al. Molecular analysis of the short arm of chromosome 3 in small-cell and non-small-cell carcinoma of the lung. N Engl J Med 1987; 317(18):1109-13.

## Kok K, Osinga J, Carritee B, et al. Deletion of a DNA sequence at the chromosomal region 3p21 in all major types of lung cancer. Nature 1987; 339:578-581.

## Devilee P, vad den Broek M, Kuipers-Dijkshoorn N, Kolluri R, Khan PM, Pearson PL and Corenelisse CJ. At least four different chromosomal regions are involved in loss of heterozygoisty in human breast carcinoma. Genomics 1989; 5:554-560.

## Lothe RA, Fossa SD, Stenwig AE, Nakamura Y, White R, Borrensen AL, and Brogger A. Loss of 3p and 11p alleles is associated with testicular cancer tumors. Genomics 1989; 5:134-138.

## Yokota J, Tsukada Y, Nakajima T, et al. Loss of heterozygosity on the short arm of chromosome 3 in carcinoma of the uterine cervix. Cancer Res. 1989; 49:3598-3601.

## Ehlen T, and Dubeau L. Loss of heterozygoisty of chromosomal segments 3p, 6q, and 11p in human ovarian carcinomas. Oncogene 1990; 5:2-9.

## Killary AM, Wolf E, Giambernardi T, Naylor SL. Definition of a tumor suppressor gene within human chromosome 3p21-p22. Proc Natl Acad Sci 1992; 89:10877-10881.

## 11. Sanchez el-Naggar A, Pathak S and Killary AM. A tumor suppressor locus within 3p14-p12 mediates rapid cell death of renal cell carcinoma in vivo. Proc Natl Acad Sci

U S A. (1994); 91, 3383-3387.

12. Lott ST, Lovell M, Naylor SL, and Killary AM. Physical and Functional Mapping of a Tumor Suppressor Locus for Renal Cell Carcinoma within Chromosome 3p12. *Cancer Res*. 1998; 58:3533-3537.

13. Lovell M, Lott ST, Wong P, and Killary AM. The Genetic Locus NRC-1 Within Chromosome 3p12 Mediates Tumor Suppression in Renal Cell Carcinoma Independently of Histologic Type, Tumor Microenvironment and VHL Mutation. *Cancer Res*. 1999; 59:2182-2189.

14. Zhang K, Lott ST, Jin L and Killary AM. Fine mapping of the *NRC-1* tumor suppressor locus within chromosome 3p12. Biochem. Biophys. Res. Comm. 2007, 360:531-538.

15. Hoglund M, et al. Statistical dissection of cytogenetic patterns in lung cancer reveals multiple modes of karyotypic evolution independent of histological classification. Cancer Genet Cytogenet. 2004 Oct 15; 154(2):99-109.
