# Supplementary material for: DEAR1 Is a Dominant Regulator of Acinar Morphogenesis and an Independent Predictor of Local Recurrence-Free Survival in Early-Onset Breast Cancer
Source: PLoS Med. 2009 May 5;6(5):e1000068. doi: 10.1371/journal.pmed.1000068 (PMC2673042; doi:10.1371/journal.pmed.1000068)
Supplement: Table S1 — DEAR1 genetic alterations in breast cell lines. (0.02 MB DOC) [file pmed.1000068.s008.doc]

**Table S1**

***DEAR1* Genetic Alterations in Breast Cell Lines**

**Breast Tumor/Cell Genetic Alteration Presence of Absence of**

**Line Alteration in Alteration in**

**SNP Database Control Lymphocytes**

**(Number of samples screened)**

Normal Breast

Epithelium H16N-2 None N/A N/A

21PT Codon 187 mutation No 136 normal

CGG→TGG,R→W alleles

21NT Codon 187 mutation No 136 normal

CGG→TGG,R→W alleles

21MT Codon 187 mutation No 136 normal

CGG→TGG,R→W alleles

MDAMB468 G→A Intron Nt 28 No 114 normal

ds exon 2 alleles
