# Supplementary material for: DEAR1 Is a Dominant Regulator of Acinar Morphogenesis and an Independent Predictor of Local Recurrence-Free Survival in Early-Onset Breast Cancer
Source: PLoS Med. 2009 May 5;6(5):e1000068. doi: 10.1371/journal.pmed.1000068 (PMC2673042; doi:10.1371/journal.pmed.1000068)
Supplement: Table S2 — DEAR1 genetic alterations in breast tumors. (0.03 MB DOC) [file pmed.1000068.s009.doc]

**Table S2**

***DEAR1* Genetic Alterations in Breast Tumors**

**Breast Tumor/Cell Genetic Alteration Presence of Absence of**

**Line Alteration in Alteration in**

**SNP Database Control Lymphocytes**

**(Number of samples screened)**

Breast Tumor Codon 187 mutation No 136 normal lymphocyte

(S04T) CGG→CAG,R→Q alleles

Breast Tumor Codon 473 mutation No 80 normal lymphocyte

(B17T) GTC→ATC,V→1 alleles; also not patients

normal lymph node

Breast Tumor Codon 350 mutation No 138 normal lymphocyte

(K06T) GTC→G/ATC, V→V/I alleles

Breast Tumor Codon 198 silent No ND

(S03T) mutation

GAG→GAA

Breast Tumor Homozygous No N/A

(9BT) deletion

Breast Tumor G→A Intron Nt 28 No 114 normal lymphocyte

(S02T) ds exon 2 alleles

Breast Tumor G→A Intron Nt 28 No 114 normal lymphocyte

(S487T) ds exon 2 alleles

Breast Tumor G→A Intron Nt No 106 normal lymphocyte

(K09T) 12 ds exon 1 alleles
