# Supplementary material for: DEAR1 Is a Dominant Regulator of Acinar Morphogenesis and an Independent Predictor of Local Recurrence-Free Survival in Early-Onset Breast Cancer
Source: PLoS Med. 2009 May 5;6(5):e1000068. doi: 10.1371/journal.pmed.1000068 (PMC2673042; doi:10.1371/journal.pmed.1000068)
Supplement: Table S3 — Primers used to identify a homozygous deletion in breast tumors. (0.04 MB DOC) [file pmed.1000068.s010.doc]

**Table S3**

**Primers used to identify a homozygous deletion (HD) in breast tumors**

|  | Start-End* | Primer | Sequence | Amplicon |
| --- | --- | --- | --- | --- |
| MS1 | chr1:33420695-33420909 | Forward | 5’-TCCCTTATCCCCTCTCCATC-3’ | 215bp |
|  |  | Reverse | 5’-TTAAGGAGTGCTTGGGGAGA-3’ |  |
| MS2 | chr1:33418095-33418244 | Forward | 5’-GCTCAAATATCCTCTCCGTGA-3’ | 150bp |
|  |  | Reverse | 5’-CCAAGGGGGTGGGTATAAAA-3’ |  |
| MS5 | chr1:33411516-33411699 | Forward | 5’-GAAAAGCAAGGCTTGACCAG-3’ | 184bp |
|  |  | Reverse | 5’-TGCCTGCTCACATTTGTTTC-3’ |  |
| PMA1 | Chr1:33420150-33420235 | Forward | 5’-BIO-TTTYGGGTTGAGAGGTTG-3’ | 86 bp |
|  |  | Reverse | 5’-ACCCCAAACCCTAACCCA-3’ |  |
|  |  | Sequencing | 5’-CCAAACCCTAACCCAAC-3’ |  |
| PMA4 | chr1:33419182-33419265 | Forward | 5’-TGTAYGAGTAGTATTAGGTTA-3’ | 106 bp |
|  |  | Reverse | 5’-GACGGGACACCGCTGATCGTTT  ACCCCTTCCCCAAACAAACC-3’ |  |
|  |  | Sequencing | 5’-GAGTAGTATTAGGTTAT-3’ |  |

*Note: based on the March 2006 human reference sequence: NCBI Build 36.1
